# Longitudinal Changes in Brain Network Metrics and Their Correlations with Spinal Cord Diffusion Tensor Imaging Parameters Following Spinal Cord Injury and Regenerative Therapy

**DOI:** 10.3390/biomedicines13123124

**Published:** 2025-12-18

**Authors:** Ting Feng, Can Zhao, Wen-Nan Su, Yi-Meng Gao, Yuan-Yuan Wu, Wen Zhao, Jia-Sheng Rao, Zhao-Yang Yang, Xiao-Guang Li

**Affiliations:** 1 Beijing Key Laboratory for Biomaterials and Neural Regeneration, National Medical Innovation Platform for Industry-Education Integration in Advanced Medical Devices (Interdiscipline of Medicine and Engineering), School of Biological Science and Medical Engineering, Beihang University, Beijing 100191, China; fengt@buaa.edu.cn (T.F.); n19834278789@163.com (W.-N.S.); gym_o_summer@buaa.edu.cn (Y.-M.G.); yuanywu@buaa.edu.cn (Y.-Y.W.); 2 Beijing International Cooperation Bases for Science and Technology on Biomaterials and Neural Regeneration, Beijing Advanced Innovation Center for Biomedical Engineering, Beihang University, Beijing 100191, China; 3 Institute of Rehabilitation Engineering, China Rehabilitation Science Institute, Beijing 100068, China; zhaocan05@163.com; 4 Department of Neurobiology, School of Basic Medical Sciences, Capital Medical University, Beijing 100069, China; wensucc@163.com

**Keywords:** spinal cord injury, structural covariance network, functional network, global metrics, spinal cord diffusion tensor imaging

## Abstract

**Objectives:** Spinal cord injury (SCI) disrupts the microstructure of the spinal cord, triggers reorganization of the brain network, and causes motor deficits. However, the temporal dynamics and interrelationships of these alterations remain unclear. **Methods:** Eight monkeys underwent spinal cord hemisection and were randomly assigned to either the SCI-only group or the treatment group that received neurotrophin-3-chitosan implants. Longitudinal brain structural/resting-state magnetic resonance imaging and spinal cord diffusion tensor imaging (DTI) were conducted. Concurrently, hindlimb motor function was assessed. The brain network topology was characterized through graph theory. The generalized additive mixed model (GAMM) was employed to analyze the longitudinal trajectories of network metrics, while the linear mixed-effects model (LMM) was used to evaluate the moderating effect of treatment on correlations between network metrics and motor/DTI parameters. **Results:** The SCI-only group exhibited sustained functional network segregation, aberrant structural topology, and lower fractional anisotropy (FA). These findings collectively reflect chronic maladaptive plasticity. In the treatment group, the therapy not only enhanced white matter integrity, reflected by increased FA values, but also reduced the clustering coefficient (Cp) in brain structural network, indicating a shift away from maladaptive segregation. Critically, the LMMs further revealed that treatment moderated the pathological correlations between global efficiency (Eg), local efficiency, Cp, and locomotor parameters. Moreover, spinal FA exerted a significant main effect on Eg of brain functional networks. **Conclusions:** These findings suggest that treatment-induced brain reorganization underlies motor function following SCI, and progressive brain reorganization correlates with changes in spinal cord microstructure, revealing a systems-level mechanism of neural repair.

## 1. Introduction

Spinal cord injury (SCI) disrupts the structural and functional integrity of the entire central nervous system, leading to brain reorganization and motor function impairment [1,2]. While structural magnetic resonance imaging (MRI) and resting-state functional magnetic resonance imaging (rs-fMRI) have demonstrated effectiveness in examining changes in grey matter volume (GMV), thickness, and functional connectivity (FC) following SCI [3,4,5,6,7,8], these methods offer limited insight into global network-level topology.

Graph theory provides a robust framework to quantify brain network topology in disease research [9,10,11]. However, to the best of our knowledge, the majority of prior SCI investigations have primarily centered on alterations in the functional network [12,13,14]. Several studies have even documented non-significant differences in global metrics between SCI patients and their healthy counterparts [15,16], with potential variables including the type of SCI, injury duration, and lesion location. These inconsistencies highlight a significant void: there is a pressing need for an in-depth, longitudinal examination of whole-brain network reorganization post-SCI.

The structural covariance network (SCN) refers to the covariance among inter-regional structural MRI markers. While SCN analysis has been extensively utilized in the research of neurodegenerative and psychiatric disorders [17,18], its use in studying SCI is relatively limited. A recent study, however, revealed that changes in the SCN of adults with SCI significantly contrast the direction of changes observed during developmental stages, thereby underscoring the need for further longitudinal exploration [19].

While spontaneous recovery is often limited, the primary therapeutic goal is to foster neural plasticity and restore motor function [20]. Our team has previously demonstrated that neurotrophin-3 (NT3)-chitosan biomaterials can promote functional recovery by stimulating endogenous neural stem cells and re-establishing synaptic connections across lesion sites [21,22]. However, it is still unclear whether and how these treatments affect the relationship between brain network reorganization and motor outcomes. The link between spinal cord integrity and brain network dynamics remains similarly elusive.

We propose that both spontaneous recovery and NT3-chitosan treatment will lead to distinct alterations in global network metrics, which will have correlations with motor performance and spinal cord diffusion tensor imaging (DTI) parameters. To this end, we integrated graph theory metrics with longitudinal MRI data to map the spatiotemporal dynamics of network reorganization following SCI and treatment. We assessed the association between spinal cord microstructural integrity and brain network reorganization. Subsequently, we delineated the longitudinal trajectories of brain network metrics post-SCI and post-treatment. Lastly, we deployed linear mixed-effects models to quantitatively evaluate whether regenerative therapy significantly moderates the relationship between brain network metrics and hindlimb locomotor parameters.

## 2. Materials and Methods

### 2.1. Spinal Cord Injury Modeling

This study involved eight adult female rhesus monkeys, averaging a weight of 5 ± 1 kg and an age range of 5–6 years. The monkeys were divided equally into two groups: the SCI-only group and the treatment group. The surgical procedure for inducing SCI was carried out as detailed in previous studies [21,22,23]. Prior to surgery, anesthesia was induced via muscle injection of ketamine hydrochloride solution (10 mg/kg, Provet, Dubai, United Arab Emirates) and xylazine hydrochloride (5 mg/kg, GlpBio, Montclair, CA, USA). This was then sustained by intravenous infusion of pentobarbital sodium (20 mg/kg, New Asia Pharmaceutical, Shanghai, China). The right-sided spinal cord injury at the T7–T9 thoracic vertebrae was executed using laminectomy, which entailed the removal of a 10 mm long and 2–3 mm wide segment of spinal cord tissue. Following local hemostasis, an appropriately sized repair material was implanted into the injury area of NT3-treated rhesus monkeys. In contrast, rhesus monkeys with only SCI did not receive any implanted materials. The dura mater, muscles, and skin were subsequently sutured sequentially. Postoperative care included daily injections of antibiotics (penicillin 240 mg/day, Heowns, Tianjin, China) and analgesics (Pentazocine 2 mg/kg, CR Double-Crane, Beijing, China) for five days. This experimental protocol has been approved by the Biomedical Ethics Committee of Beihang University (BM20240014). All animal housing and experiments were conducted in strict accordance to the institutional guidelines for care and use of laboratory animals.

### 2.2. MRI Protocol

All data were collected on a 3T Siemens MRI system (Magnetom Skyra, Siemens, Erlangen, Germany). Scans were conducted during the healthy period (baseline) and at 1, 2, 3, 6, and 12 months after SCI. Prior to scanning, anesthesia was induced by intramuscular injection of ketamine hydrochloride solution (10 mg/kg, Provet, Dubai, United Arab Emirates), and atropine sulfate (0.05 mg/kg, Jinyao Pharmaceutical, Tianjin, China) was administered intramuscularly to reduce salivation. Anesthesia was maintained using a mixture of propofol (0.25 mg/kg/min, Libang Pharmaceutical, Xian, China) and ketamine (0.03 mg/kg/min, Provet, Dubai, United Arab Emirates) in saline for intravenous infusion.

Imaging data of brain were acquired using a customized four-channel primate head transmitter receiver coil. All animals were placed in the prone position. The rs-fMRI data were acquired using a gradient echo-echo planar imaging sequence (GRE-EPI) with the following parameters: repetition time (TR)/echo time (TE) = 2000 ms/30 ms; field of view (FOV) = 128 × 128 mm ^2^; matrix = 64 × 64; slice thickness = 2 mm; flip angle = 90°; number of slices = 25; scan time = 4 min, and 120 volumes of data were obtained.

T1-weighted imaging structural data were acquired using a three-dimensional (3D) magnetization-prepared rapid gradient echo sequence (TR/TE = 1520 ms/4.42 ms; flip angle = 15°; inversion time (TI) = 520 ms; slices = 240, continuous slice voxel size = 0.5 mm^3^).

Imaging data of spinal cord were acquired using a 32-channel receive-only array spine coil. All animals were positioned in the supine position. Spinal cord structural images were acquired with a proton-density weighted sequence using the following parameters: TR = 3050 ms; TE = 11 ms; flip angle = 15°; matrix = 320 × 320; and resolution = 0.6 × 0.6 × 2.0 mm^3^. The scanning center was aligned with the spinal cord surgical site, 27 consecutive axial slices were obtained. For diffusion tensor images of spinal cord, a single-shot spin-echo echo-planar imaging (SE-EPI) sequence was applied with the following parameters: TR = 4500 ms, TE = 104 ms, matrix size = 128 × 128, FOV = 196 × 196 mm, resolution = 1.5 × 1.5 × 2.0 mm^3^, and number of excitations (NEX) = 6. Diffusion weighting was applied using b-values of 0 and 1000 s/mm^2^ along 12 gradient directions. The scanning center was aligned with the spinal cord surgical site, capturing 25 consecutive slices that encompassed both the operative region and adjacent healthy tissue at rostral and caudal levels.

### 2.3. Preprocessing of MRI Data

The rs-fMRI data preprocessing was performed using the resting-state fMRI data processing software (http://restfmri.net/forum/DPARSF, version 5.1, accessed on 1 December 2020). Specifically, firstly, the first 10 volumes of each functional time series were removed. Secondly, slice timing correction was performed. Head motion was assessed and corrected using rigid-body registration, excluding datasets where maximum translation exceeded 2 mm and maximum rotation exceeded 2°. Third, we normalized the corrected fMRI image space to the INIA19 non-human primate brain atlas. Fourth, each voxel was resampled to an isotropic 0.5 × 0.5 × 0.5 mm^3^. Fifth, the images were smoothed using a 6-mm Gaussian kernel. Sixth, we regressed out nuisance covariates, including global signals and signals from white matter (WM) and cerebrospinal fluid (CSF). Seventh, a band-pass filter (0.01–0.1 Hz) was used to reduce low-frequency drift and high-frequency noise.

The T1-weighted imaging data were preprocessed using SPM8. We used the tissue probability maps (TPM) constructed by Rohlfing et al. [24] for macaques to segment the images into gray matter (GM), WM, and CSF. The segmented images during the healthy period were used to create macaque GM and WM specific templates using a Diffeomorphic Anatomical Registration Through Exponentiated Lie Algebra (DARTEL)-based method. Individual GM images registered to the template were modulated by multiplying the voxel values by the Jacobian determinant of the deformation field. The final specific template was spatially normalized to the atlas space through affine transformation, and this transformation field was used to spatially normalize the modulated GM images to the atlas space. The generated images were smoothed using an isotropic Gaussian kernel with a full width at half maximum of 2 mm. All magnetic resonance imaging data underwent uniform, batch processing using identical parameters. The aforementioned rigorous processing procedures ensured that no missing values were present for any included macaques; consequently, all data were used for network construction and analysis.

### 2.4. Functional Network and Structural Covariance Network Construction

For construction of functional network, we first divided the entire brain according to the INIA19 primate brain atlas [24]. The time series in 470 regions of interest (ROI) was obtained by averaging the time series of all voxels within each ROI. The edge of the correlation matrix was measured by calculating the Pearson correlation coefficients between the preprocessed time series of each pair of ROIs. Given the ambiguous interpretation of negative correlations, this analysis was conducted only for positive correlations [25]. Next, the Pearson correlation coefficient between each pair of nodes was converted into Z-scores, in order to perform statistical analysis. Therefore, for each animal, symmetric positive FC matrices were obtained.

The specific steps for constructing structural covariance networks are as follows: First, a linear regression model was used to adjust the regional GMV values for covariates, and the total intracranial volume [26], and then the GMV residuals of the brain regions were extracted. Next, by calculating the Pearson correlation coefficient between the residuals in brain regions, the group-level structural covariance matrices during the healthy period were constructed. The average $M_{HC,i}$ and the standard deviation ${SD}_{HC,i}$ of GMV during the healthy period were calculated. The interregional effect size difference (ESD) for each rhesus monkey at each time point was calculated according to Formula (1). The correlation coefficient R was acquired using Formula (2). The individual weighting matrix $W\left(i,j\right)$ for each rhesus monkey was calculated using Formula (3) [27]. The individual SCN matrix $M\left(i,j\right)$ was calculated by element-wise multiplication between the individual weighting matrix with the group-level structural covariance matrix using Formula (4).

ESD(i,j), R(i,j), $W\left(i,j\right)$ and $M\left(i,j\right)$ are defined as follows:

(1) $$ESD\left(i,j\right)=\;\frac{\left|\left(x_{i}-\;M_{HC,i}\right)-\left(x_{j}-\;M_{HC,j}\right)\right|}{\sqrt{\frac{{SD}_{HC,i}^{2}+{SD}_{HC,j}^{2}}{2}}}$$

where $x_{i}$ and $x_{j}$ be regional GMV values for regions *i* and *j* from each macaque.

(2) $$R(i,j)=\frac{\exp\left(2\times ESD\left(i,j\right)\right)-1}{\exp\left(2\times ESD\left(i,j\right)\right)+1}$$

(3) $$W\left(i,j\right)=1-R(i,j)$$

(4) $$M\left(i,j\right)=W\left(i,j\right).\;*\;{SCN}_{HC}(i,j)$$

where ${SCN}_{HC}\left(i,j\right)$ be the group-level structural covariance matrix during the healthy period.

### 2.5. Calculation of Global Metrics and Their Area Under the Curve in Brain Network

Some have argued that weighted graphs are not biologically plausible, as brain regions typically form sparse anatomical connections only with specific other regions [28]. To define binary graphs, researchers typically apply either an absolute (correlation-based) or a proportional (sparsity-based) threshold. When comparing groups, applying a proportional threshold enables more effective comparison of network metrics across them. The use of proportional thresholds improves the stability of network measures [29]. To compare between-group differences group under the same cost conditions, the sparsity threshold range from 0.05 to 0.5 (in increments of 0.01) was applied to functional and structural covariance matrix. This specific sparsity range aligns with established practices in brain network research [16,30,31,32] and ensured that all constructed networks maintained small-world properties, a fundamental characteristic of brain organization. The upper limit of 50% was set for two reasons: to preserve small-world topology, and because networks exceeding this density are considered biologically implausible and of limited neurobiological relevance [33,34]. In this study, we used functions from the Graph Theoretical Network Analysis (GRETNA) package to measure the global metrics of the functional and structural covariance network within this threshold range, including global efficiency (Eg), local efficiency (Eloc), characteristic path length (Lp), and clustering coefficient (Cp), small-worldness (σ), normalized clustering coefficient (γ), characteristic path length (λ). We also calculated the area under the curve (AUC) of each metric within this specific threshold for statistical analysis. The AUC provides a comprehensive scalar for network metrics and offers a sensitive method for detecting abnormalities in brain networks in diseases [35].

### 2.6. Calculation of Spinal Cord DTI Parameters

The spinal cord diffusion tensor images were processed using the Spinal Cord Toolbox (SCT, version 6.4) [36]. First, the images were cropped around the spinal cord centerline, followed by motion and eddy current corrections to generate the mean b0 images, mean diffusion weighted images, and corrected DTI datasets. To correct geometric distortion, the mean b0 image was registered to the proton density-weighted volume, yielding a nonlinear deformation vector field [37]. This field was subsequently applied to all echo-planar imaging datasets [38]. Tensor fitting of the corrected data yielded fractional anisotropy (FA), radial diffusivity (RD), mean diffusivity (MD), and axial diffusivity (AD) maps. The ROIs were drawn to cover the entire spinal cord cross-section on three consecutive axial slices at the lesion epicenter. Selecting a single slice risks deviation from the true lesion epicenter, potentially missing the injury core and yielding unreliable results. Therefore, to improve robustness and reduce this selection bias, we applied a three-layer averaging method. The ITK-SNAP (version 3.6.0) was used to manually trace the ROI on the FA images. To minimize partial volume averaging with CSF, the ROI was positioned at least one voxel away from the spinal cord border [39]. All DTI metrics were then extracted from the defined ROI.

### 2.7. Hindlimb Locomotor Function Evaluation

To assess the motor function, the gait trajectory of the right hindlimb was recorded when rhesus monkeys walking on the treadmill bipedally with restraining upper body. The Vicon system (Oxford Metrics Limited Company, Dayton, UK) was used to capture motor performance. The path length, step height and stride length were extracted. The treadmill speed was set to 0.5 m/s.

### 2.8. Longitudinal Trajectories of Global Metrics

To estimate longitudinal changes in global metrics over time in both the SCI-only group and the treatment group, we next applied the generalized additive mixed model (GAMM) in R (version 4.4.1) through the mgcv package. The duration of injury was included as a smoothing term, and subject ID (subID) was included as a random effect to account for individual differences. Nonlinearity is estimated using the restricted maximum likelihood method to prevent data overfitting [40]. The smoothing parameter k is determined by comparing the Bayesian information criterion (BIC) values to optimally balance the fit of all variables.

### 2.9. Linear Mixed-Effects Models Examining the Interaction of Brain Network Metrics and Group on Locomotor Parameters

To examine the relationship between brain network metrics and locomotor parameters after SCI, and to assess potential differences between the SCI-only and treatment groups, we applied linear mixed-effects models (LMMs) using the lme4 package in R (version 4.4.1). The association was evaluated in two ways: first, using global metrics at a fixed sparsity threshold of 0.1, consistent with prior work [41], and second, using the AUC computed for each global metric across a sparsity range of 0.05 to 0.50. In these models, locomotor parameters served as the dependent variable. Fixed effects comprised the brain network metrics, group, their interaction, and time point, with the interaction term being of primary interest to test whether the brain–motor relationship differed by group. Random intercepts for subID accounted for repeated measurements. Denominator degrees of freedom and F-statistics for fixed effects were estimated via the Kenward-Roger method, which improves error rate control and model robustness, particularly with smaller sample sizes.

When a significant interaction emerged, we conducted post hoc analyses to interpret its nature. Simple slope analysis via the “emmeans” package evaluated the brain network metrics–locomotor parameters association within each group, with inference based on the Kenward-Roger method. We then directly compared these slopes between groups. All analyses were performed in R (version 4.4.1) using the “lme4”, “lmerTest”, and “emmeans” packages. We evaluated model performance using marginal and conditional R^2^ to quantify the overall explained variance [42]. Cohen’s f^2^ was calculated to assess the effect size of the interaction terms [43,44].

### 2.10. Linear Mixed-Effects Models Examining the Interaction of Spinal Cord DTI Parameters and Group on Brain Network Metrics

We also used LMMs to assess the association between spinal cord DTI parameters and brain network metrics. These models tested for group differences while including time point as a covariate. Random intercepts for subject ID were incorporated to address within-subject correlation across repeated assessments. Fixed effects included spinal cord DTI parameters, group, time point, and the interaction between DTI parameters and group. Models were fitted with restricted maximum likelihood (REML), and degrees of freedom were again determined using the Kenward-Roger approximation.

### 2.11. Statistical Analysis

For between-group comparisons (treatment vs. SCI-only) at each time point, an independent samples *t*-test was applied if the data in both groups satisfied normality and homogeneity of variance. Welch’s *t*-test was used if the data were normally distributed but variances were unequal. The non-parametric Mann–Whitney U test was employed if the data from either group deviated from normality. For within-group comparisons between each follow-up time point and baseline, a paired samples *t*-test was used. If the paired differences deviated from normality, the Wilcoxon signed-rank test was utilized instead. A statistically significant difference was defined as *p* < 0.05. A flowchart summarizing the entire workflow from data preprocessing to network construction and statistical analysis is provided in Figure S1.

## 3. Results

In the current study, group differences were tested for all global metrics of functional network and the SCN across a range of network sparsity thresholds (0.05:0.01:0.50). Healthy baseline showed typical small-worldness (γ >> 1, λ ≈ 1, σ > 1) across the entire range of sparsity thresholds, indicating a high clustering and short path length of the brain network [45,46], and thus the potential for functional specialization or modular processing, as well as distributed or integrated processing across the entire network [47].

### 3.1. SCI Facilitates the Integration of Functional Brain Network

**Figure 1:**
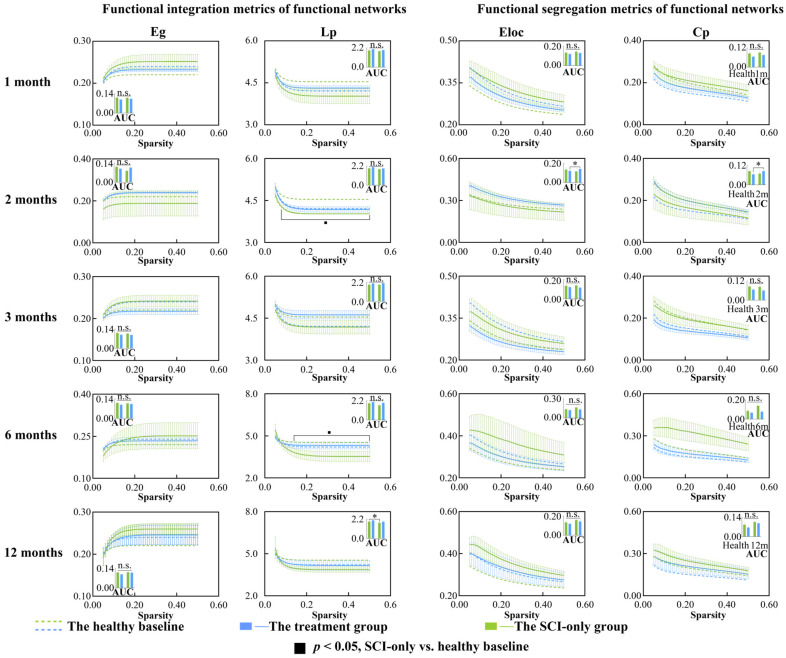
Between-group and within-group differences in functional integration and segregation metrics within functional network, including Eg, Lp, Eloc and Cp. Solid squares represent significant differences between the SCI-only group and its healthy baseline. Open squares represent significant differences between the treatment group and its healthy baseline. Solid circles represent significant differences between the SCI-only group and the treatment group. A *p*-value less than 0.05 is considered to indicate significant between-group differences. n.s., no significance. * *p* < 0.05. Eg, global efficiency; Lp, characteristic path length; Eloc, local efficiency; Cp, clustering coefficient.

We first examined global metrics of functional networks between and within groups in the first year after SCI and its treatment. There were no significant differences in small-world characteristics of the functional networks (Figure S2). For functional integration (Figure 1), at different sparsity thresholds ranging from 0.08 to 0.50, Lp was significantly lower than baseline in SCI-only group at 2 and 6 months post-injury (*p*s < 0.50). At 12 months post-SCI, the AUC of Lp (*p* = 0.041) in SCI-only group was also significantly lower than baseline. In terms of functional segregation metrics, the AUCs of Eloc (*p* = 0.039) and Cp (*p* = 0.035) in the treatment group at 2 months post-SCI were significantly higher than baseline. For more detailed statistical results including *p*-values, t-statistic, confidence intervals, and effect sizes, see Table S1.

### 3.2. Regenerative Therapy Reverses the Properties of SCN Disrupted by SCI

**Figure 2:**
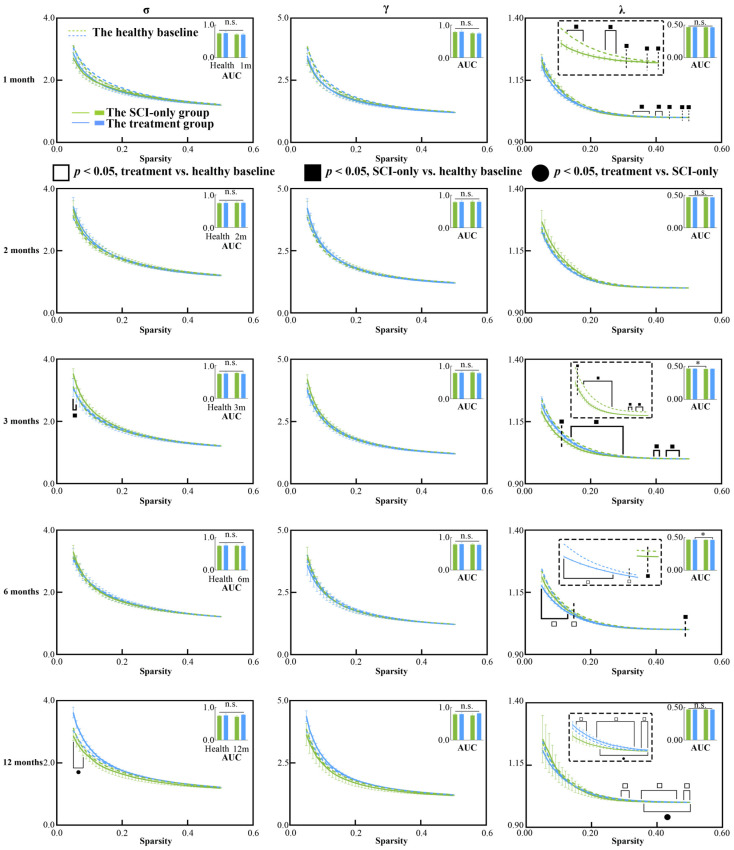
Between-group and within-group differences in small-world characteristics within structural covariance network, including σ, γ and λ. Solid squares represent significant differences between the SCI-only group and its healthy baseline. Open squares represent significant differences between the treatment group and its healthy baseline. Solid circles represent significant differences between the SCI-only group and the treatment group. A *p*-value less than 0.05 is considered to indicate significant between-group differences. n.s., no significance. * *p* < 0.05. γ, normalized clustering coefficient; λ, normalized characteristic path length; σ, small-worldness.

**Figure 3:**
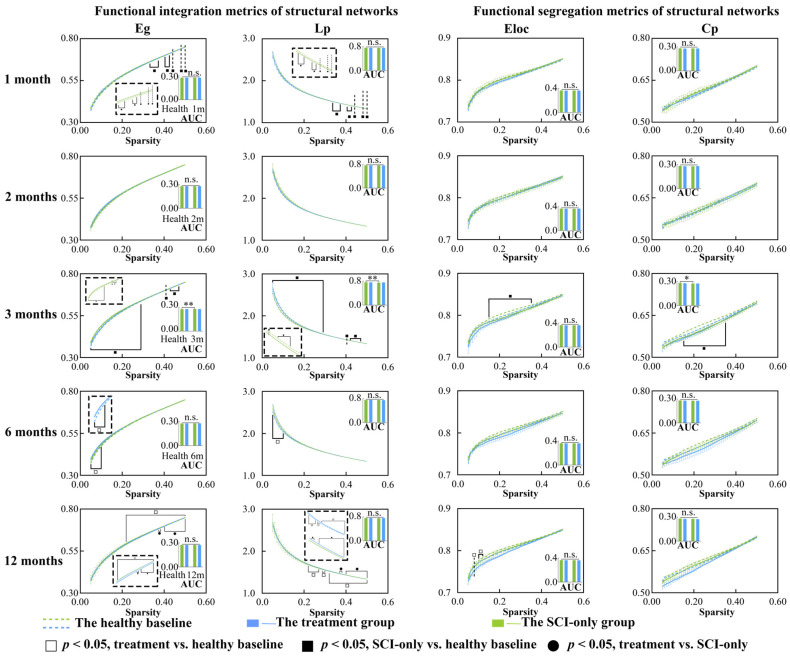
Between-group and within-group differences in functional integration and segregation metrics within structural covariance network, including Eg, Lp, Eloc and Cp. Solid squares represent significant differences between the SCI-only group and its healthy baseline. Open squares represent significant differences between the treatment group and its healthy baseline. Solid circles represent significant differences between the SCI-only group and the treatment group. A *p*-value less than 0.05 is considered to indicate significant between-group differences. n.s., no significance. * *p* < 0.05, ** *p* < 0.01. Eg, global efficiency; Lp, characteristic path length; Eloc, local efficiency; Cp, clustering coefficient.

We then assessed changes in global metrics of the SCN after SCI (Figure 2 and Figure 3). Temporal and treatment-related changes were evident in both network integration and segregation metrics. In the SCI-only group, the AUC of λ (*p* = 0.010) was significantly reduced compared to baseline at the 3 months. λ values at several sparsity thresholds ranging from 0.11 to 0.50 were significantly lower than the healthy at 1, 3 and 6 months after injury (*p*s < 0.50), indicating a widespread and persistent reduction in global integration. Moreover, the SCI-only group showed significantly greater σ at sparsity thresholds of 0.05 and 0.06 three months after injury (*p*s ≤ 0.044). The treatment group exhibited a biphasic response in λ. At 6 months, both the AUC of λ (*p* = 0.0496) and λ values at low sparsity thresholds (0.015–0.13) were significantly decreased (*p* ≤ 0.049). However, at 12 months, substantial recovery was seen as λ was significantly increased compared to its own baseline (*p*s ≤ 0.046) and the SCI-only group (*p*s ≤ 0.049) at high sparsity thresholds between 0.36 and 0.50.

With respect to the integration metrics, in comparison with the healthy baseline, the SCI-only group showed significantly decreased Lp (*p*s ≤ 0.049) and increased Eg (*p*s ≤ 0.049) across sparsity thresholds of 0.33 to 0.50 at 1 month. These effects were more pronounced at 3 months, where the AUC of Eg was significantly increased (*p* = 0.003), and Eg (*p*s ≤ 0.043) was still higher than the baseline across almost the entire sparsity range (0.05–0.47), while both the AUC of Lp (*p* = 0.004) and its values over the range of 0.05–0.47 were significantly lower than the baseline (*p*s ≤ 0.043). At 6 months, the treatment group showed signs of emerging reorganization, having significantly higher Eg (*p*s ≤ 0.041) and significantly lower Lp (*p*s ≤ 0.044) than the healthy baseline over the low sparsity range of 0.05 to 0.10. This trend was reversed at high sparsity thresholds (0.22–0.50) at 12 months, where Eg was significantly lower than the healthy baseline (*p*s ≤ 0.041) and Lp was significantly higher compared to both its own baseline (*p*s ≤ 0.042) and the SCI-only group (*p*s ≤ 0.049), especially over the range of 0.37–0.50.

In examining network segregation metrics relative to their own healthy baseline, the SCI-only group demonstrated a significant decrease in the AUC of Cp (*p* = 0.013) at 3 months, with both Cp (*p*s ≤ 0.042) and Eloc (*p*s ≤ 0.042) significantly decreased across sparsity thresholds ranging from 0.15 to 0.35. At 2 months, the treatment group also exhibited increased Cp (*p* = 0.027) compared to the healthy baseline at sparsity threshold of 0.07. At 12 months, the treatment group also exhibited reduced Eloc (*p*s ≤ 0.045) compared to the healthy baseline, though specifically within the low sparsity range of 0.08 to 0.12. All *p*-values, t-statistics, confidence intervals, effect sizes, and other relevant statistical information can be found in Tables S2 and S3.

### 3.3. Regenerative Therapy Preserves the Integrity of Spinal Cord Microstructure

**Figure 4:**
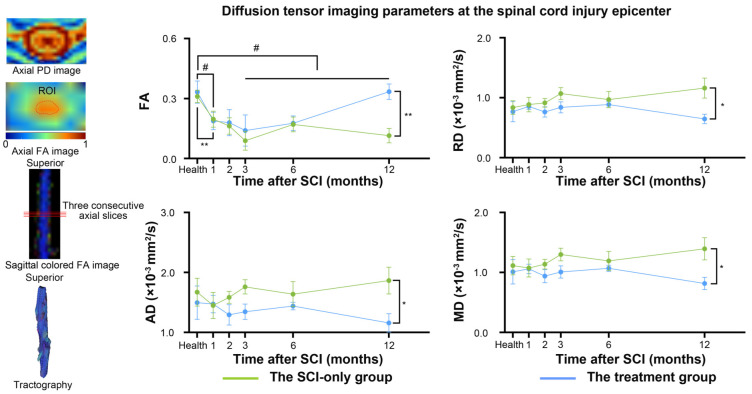
Between-group and within-group differences in spinal cord diffusion tensor imaging parameters. # represents significant differences between the SCI-only group and its healthy baseline. A *p*-value less than 0.05 is considered to indicate significant between-group differences. n.s., no significance. * *p* < 0.05, ** *p* < 0.01. FA, fractional anisotropy; RD, radial diffusivity; MD, mean diffusivity; AD, axial diffusivity.

DTI parameters were measured in the entire spinal cord on three consecutive axial slices through the injury epicenter (Figure 4). Analysis of FA revealed that, in the SCI-only group, FA values were significantly lower than the baseline at 1, 3, 6, and 12 months after SCI (1 month, *p* = 0.032; 3 months, *p* = 0.014; 6 months, *p* = 0.005; 12 months, *p* = 0.023). In the treatment group, FA was also significantly reduced compared to baseline at 1 month after SCI (*p* = 0.007). At 12 months post-injury, a significant protective effect of the treatment on tissue microstructure was observed as demonstrated by significantly increased FA (*p* = 0.006) and significantly decreased MD (*p* = 0.034), RD (*p* = 0.032), and AD (*p* = 0.041) in the treatment group compared to the SCI-only group at the injury epicenter. For specific t-statistic, confidence intervals, effect sizes, and other details, see Tables S4 and S5.

### 3.4. The Integrity of Spinal Cord Microstructure Is Associated with Partial Brain Network Properties

**Figure 5:**
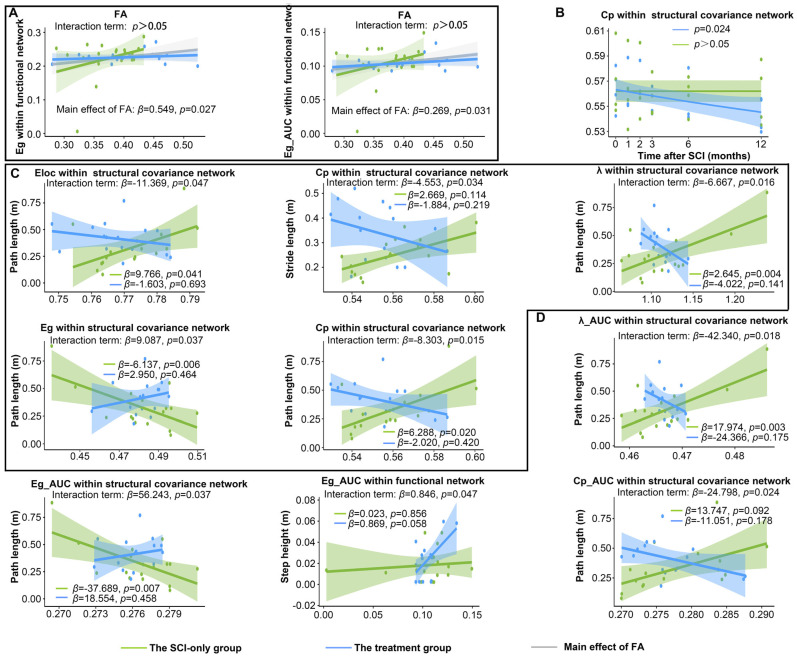
Longitudinal changes in brain network metrics and their associations with locomotor and spinal cord DTI parameters after SCI and treatment. (**A**) Main effects of FA on brain network metrics. (**B**) Significant longitudinal variation in global metrics with the duration of injury. (**C**) The moderating effect of treatment on the relationship between global metrics at sparsity threshold of 0.1 and hindlimb locomotor parameters. (**D**) The moderating effect of treatment on the relationship between the AUC of global metrics and hindlimb locomotor parameters. λ, normalized characteristic path length; Eg, global efficiency; Lp, characteristic path length; Eloc, local efficiency; Cp, clustering coefficient; FA, fractional anisotropy.

In addition, to investigate the association between the integrity of white matter microstructure in the spinal cord and global metrics of brain networks, as well as to determine whether treatment influenced this relationship, we applied linear mixed-effects models with time point as the fixed factor and subID as the random intercept (Figure 5A). No significant interaction effect was detected, but a significant main effect of FA was revealed. In the model exploring the relationship between FA and Eg of functional networks, the interaction term was not statistically significant (*β* = −0.570, 95% CI [−1.19, −0.001], SE = 0.299, *p* = 0.066). However, simple slope analysis indicated group-specific associations: FA exhibited a significant positive correlation with Eg in the SCI-only group (β = 0.549, 95% CI [0.043, 1.055], SE = 0.249, *p* = 0.034), but not in the treatment group (*β* = −0.021, 95% CI [−0.376, 0.335], SE = 0.175, *p* = 0.906). A significant positive main effect of FA on Eg was observed when combining data across groups (*β* = 0.549, 95% CI [0.085, 1.013], SE = 0.237, *p* = 0.027). An equivalent model computing the AUC of Eg within functional networks showed similar results. The interaction was again non-significant (*β* = −0.265, 95% CI [−0.562, 0.031], SE = 0.151, *p* = 0.088). A significant positive association between FA and the AUC of Eg was present in the SCI-only group (*β* = 0.269, 95% CI [0.013, 0.524], SE = 0.126, *p* = 0.040) but not in the treatment group (β = 0.003, 95% CI [−0.177, 0.183], SE = 0.088, *p* = 0.972). A significant positive main effect of FA on the AUC of Eg was confirmed across all animals (*β* = 0.269, 95% CI [0.034, 0.503], SE = 0.120, *p* = 0.031). No significant main effects were found for group or time point. Detailed statistics for the LMMs, simple slope analysis, and pairwise comparisons are provided in Tables S6–S8. Table S9 shows the variance explained and effect sizes of the models.

### 3.5. Regenerative Therapy Continuously Reduces the Cp of SCN

We then explored the longitudinal changes in global metrics of the network over time after SCI and post-treatment (Figure 5B). After NT3 treatment, at a network sparsity threshold of 0.10, the Cp within the SCN exhibited a significant decrease over time (*p* = 0.023). The longitudinal curves demonstrating significant changes under other network sparsity thresholds are provided in Figure S3. Furthermore, although statistically significant longitudinal trajectories for other global metrics were not obtained at a network sparsity of 0.1, trends of change were still observed (Figure S4). The Eloc within the SCN in the treatment group displayed a slight downward trend over time, which was not statistically significant. For the SCI-only group, neither the Eloc nor the Cp of the functional network showed statistical significance; however, the longitudinal curve with the duration of injury demonstrated a slight increase, potentially due to sample size limitations. Similarly, a previous study found that patients with SCI who did not experience neuropathic pain exhibited decreased Cp and Eloc, whereas those who experienced pain showed increased Cp and Eloc [48].

### 3.6. SCI and Treatment Reconfiguring the Relationship Between Global Metrics and Locomotor Performance

We next employed the LMM to determine whether post-SCI treatment moderated the relationships between global network metrics and hindlimb locomotor parameters across the post-injury period (Figure 5C). These analyses identified consistent, statistically significant interactions between group and several global metrics of the SCN. For path length, these interactions were between group and Eloc (*β* = −11.369, 95% CI [−21.974, −0.765], SE = 5.411, *p* = 0.047), Eg (*β* = 9.087, 95% CI [0.972, 17.203], SE = 4.141, *p* = 0.037), Cp (*β* = −8.303, 95% CI [−14.529, −2.077], SE = 3.176, *p* = 0.015), and λ (*β* = −6.667, 95% CI [−11.755, −1.579], SE = 2.596, *p* = 0.016). There was also a significant interaction between group and Cp for stride length (*β* = −4.553, 95% CI [−8.538, −0.567], SE = 2.033, *p* = 0.034).

The simple slopes analysis revealed that the associations of network metrics with locomotor recovery in SCI-only and treatment groups had opposite trends. In the SCI-only group, path length was significantly positively associated with Eloc (*β* = 9.766, 95% CI [0.473, 19.060], SE = 4.280, *p* = 0.041), Cp (*β* = 6.288, 95% CI [1.220, 11.360], SE = 2.280, *p* = 0.020), and λ (*β* = 2.645, 95% CI [0.939, 4.350], SE = 0.831, *p* = 0.004) and negatively associated with Eg (*β* = −6.137, 95% CI [−9.839, −2.435], SE = 1.889, *p* = 0.006). However, these associations tended to be negative in the treatment group (Eloc: *β* = −1.603, 95% CI [−9.876, 6.670], SE = 4.020, *p* = 0.693; Eg: *β* = 2.950, 95% CI [−5.200, 11.100], SE = 3.970, *p* = 0.464; Cp: *β* = −2.020, 95% CI [−7.090, 3.060], SE = 2.450, *p* = 0.420; λ: *β* = −4.022, 95% CI [−9.457, 1.410], SE = 2.650, *p* = 0.141). For stride length, the SCI-only group exhibited a positive trend for Cp (*β* = 2.669, 95% CI [−0.106, 5.444], SE = 1.416, *p* = 0.083), while this association was demonstrated as a non-significant negative trend in the treatment group (*β* = −1.884, 95% CI [−4.970, 1.202], SE = 1.489, *p* = 0.219).

To characterize generalized and threshold-independent associations, the AUC was computed for each global metric over the sparsity range 0.05–0.50. Regenerative therapy significantly modulated the relationship between AUC values of multiple brain network metrics within the SCN and locomotor parameters (Figure 5D). In the case of path length, the analysis showed significant interactions for the AUC of Eg (*β* = 56.243, 95% CI [6.160, 106.327], SE = 25.553, *p* = 0.037), Cp (*β* = −24.798, 95% CI [−44.978, −4.618], SE = 10.296, *p* = 0.024), and λ (*β* = −42.340, 95% CI [−75.258, −9.422], SE = 16.795, *p* = 0.018). Among functional networks, a significant interaction between group and the AUC of Eg was observed for step height (*β* = 0.846, 95% CI [0.075, 1.617], SE = 0.393, *p* = 0.047).

The simple slopes analysis further confirmed that the treatment modified these associations. In the SCI-only group, path length had a positive trend with the AUC of Cp (*β* = 13.747, 95% CI [0.472, 27.022], SE = 6.773, *p* = 0.092) and a significant positive association with the AUC of λ (*β* = 17.974, 95% CI [6.836, 29.112], SE = 5.422, *p* = 0.003), but a significant negative association with the AUC of Eg (*β* = −37.689, 95% CI [−60.914, −14.464], SE = 11.849, *p* = 0.004). These associations were absent in the treatment group, which instead displayed non-significant, reversed trends (the AUC of Cp: *β* = −11.051, 95% CI [−27.480, 5.378], SE = 7.990, *p* = 0.178; the AUC of λ: *β* = −24.366, 95% CI [−60.231, 11.499], SE = 17.470, *p* = 0.175; the AUC of Eg: *β* = 18.554, 95% CI [−32.100, 69.208], SE = 24.600, *p* = 0.458). For functional networks, no significant association was found between the AUC of Eg and step height in the SCI-only group (*β* = 0.023, 95% CI [−0.217, 0.264], SE = 0.122, *p* = 0.856), whereas the treatment group exhibited a strong positive trend (*β* = 0.869, 95% CI [−0.034, 1.772], SE = 0.424, *p* = 0.058).

Main effects of time point were reliable across models for path length, such that this measure increased at later time points post-injury (all *p*s ≤ 0.038). Significant main effects of group were also found in most models (*p*s ≤ 0.045); however, these effects were in different directions, indicating that the impact of treatment on motor performance varied depending on the brain network metric. Importantly, significant interaction effects between group and brain network metrics were consistently identified across models, demonstrating that the treatment fundamentally altered the association between brain network organization and motor function. Detailed statistics for the LMMs, simple slope analysis, and pairwise comparisons are provided in Tables S10–S12. Table S13 shows the variance explained and effect sizes of these models.

## 4. Discussion

This study demonstrates a multifaceted and dynamic reorganization of the brain after SCI, with an abnormally increased structural network integration indicative of a pathological, randomized topological architecture. We also observed increased functional network integration that may be part of a compensatory response to injury. Regenerative therapy modulated these changes as shown by a longitudinal decline in structural network segregation, as well as a fundamental modification in the relationship between brain network metrics and motor performance. Furthermore, the correlation between spinal cord integrity and global efficiency of the functional network indicates that functional reorganization is a compensatory response that is tightly associated with the level of microstructural damage in the spinal cord. Taken together, these findings suggest that the beneficial effects of this therapy arise not only from local restoration of tissue but also from a global modification of pathological whole-brain network reorganization toward a more adaptive state. The spatial resolution of the rs-fMRI data in this study represents a deliberate compromise between whole-brain coverage and signal-to-noise ratio, which may lead to partial volume effects. Future investigations utilizing ultra-high-field MRI to achieve sub-millimeter resolution will be important for precisely delineating fine-grained functional architectures within the macaque cortex.

### 4.1. Increased Integration of Functional Network After SCI

Significant differences were observed in the Lp of functional networks. Lp is a measure of the capacity for global communication [49]. Specifically, the SCI-only group exhibited reduced Lp at both 2 and 6 months post-injury, suggesting a pathological shift to a more random-like network topology [50,51], possibly due to the loss of long-range connections crucial for efficient information integration. This altered functional integration remained evident at 12 months, as demonstrated by a significantly reduced AUC compared to baseline.

In contrast, the treatment seemed to promote beneficial reorganization of local networks. At two months post-injury, the AUC for both Eloc and Cp was significantly higher in the treatment group compared with baseline. Theses increase indicated that the treatment enhanced fault tolerance and processing of specialized information in local brain regions, reflecting strengthened functional segregation [52,53]. This profile suggested that the treatment facilitated the reorganization of local neural circuits, thereby improving their modular processing capacity. The collective results pointed to a stepwise recovery process where the reorganization of local circuits preceded the reconstruction of global network architecture.

### 4.2. Aberrant Integration and Segregation of Structural Networks After SCI

The SCN, which is typically constructed at the group level by measuring interregional morphological similarity across a cohort, have been widely used to characterize topological organization in patients with SCI [16] and cervical spondylotic myelopathy [54]. These population-based SCNs inherently obscure interindividual variation and cannot reveal subject-specific correlations between brain topology and motor parameters. Moreover, no significant differences in the global properties of structural networks were observed between patients with complete SCI and healthy controls [16], suggesting that this group-level approach may lack sensitivity to individual pathological changes. Since previous studies have successfully implemented individual SCNs in disease research [27,55], we constructed individual SCNs based on interregional effect size differences. This method quantified the extent to which each macaque’s brain structural configuration diverged from the healthy mean baseline, thereby enabling an examination of brain-motor function relationships at the individual level.

Network integration and segregation were largely and persistently impaired in the SCN after SCI. The small-world property was defined by the criteria ≥ 1 and γ ≫ 1 [56]. Compared to the healthy baseline, SCI-only group exhibited a significant decrease in λ at 1, 3, and 6 months post-injury, suggesting a trend toward to more random network topology [57,58]. Such a deviation from an optimal small-world regime is recognized as pathological randomization which is often caused by the disruption of important long-range connections [50]. The positive correlation between λ and path length in the SCI-only group supports this viewpoint because locomotor deficits was associated with abnormal reorganization of the SCN. However, the treatment group showed a completely different result. After an initial decline at 6 months, λ increased more significantly by 12 months under high sparsity thresholds relative to the healthy baseline, indicating that the treatment fostered the progressive restoration of long-range connections and supported a more stable and integrated small-world architecture.

Significant global changes were observed in the SCI-only group at 1 month post-injury, where Lp decreased and Eg increased, indicating a pathological process of randomization. This is probably because the specialized pathways were disrupted by the loss of afferent/efferent connections [54,59,60], and the brain overly relied on increasing random-like integration as a compensatory but maladaptive mechanism. Such pathological integration was also highly stable across modalities, as it was also found in major depressive disorder [55] and DTI-based structural networks post-SCI [61]. On the other hand, the treatment group reversed this trend at 12 months, where Lp was higher and Eg was lower, suggesting that the intervention prevented further network randomization and promoted restoration of specialized topology.

Regarding network segregation, the large decreases in Cp and Eloc at 3 months in the SCI-only group suggest a disruption of local information processing, which may be associated with gray matter atrophy. The distinct decrease in Eloc at 12 months in the treatment group within the low-sparsity range may reflect an active reorganization of the network architecture, changing the disproportionate dependence on local connectivity to a more homogenous global distribution of resources.

### 4.3. Correlations Between Global Efficiency of Functional Networks and Fractional Anisotropy

FA has been shown to be a sensitive biomarker of spinal cord integrity [62,63] and motor function recovery after SCI [64,65]. Consistent with previous reports, we found significant FA decrease at 1 month post-injury in both groups, indicating severe microstructural damage initially. While the SCI-only group exhibited progressive deterioration, with further significant FA decrease and increases in RD and AD at 12 months, the treatment group demonstrated notable alleviation of these pathologies. Such changes represent ongoing white matter degradation, myelin degeneration [66], and axonal injury leading to cavitation [67] in the SCI-only group. While NT3-chitosan establishes a sustained neurotrophic supply and an anti-inflammatory microenvironment at the lesion site [68], it effectively promotes axonal regeneration and myelin repair, thereby enhancing the structural integrity of spinal cord white matter.

Furthermore, we examined the effect of this therapy on the association between spinal cord microstructure and brain network organization. While no significant interaction effect was found, spinal cord FA exhibited a significant positive main effect on the Eg of functional networks. This result indicates that maintaining spinal cord integrity is linked to better integrated whole-brain functional networks. Without this structural repair, as seen in the SCI-only group, global efficiency of brain networks remained highly dependent on residual white matter pathways in the spinal cord. In the treatment group, however, NT3-chitosan enhanced neural plasticity, resulting in newly formed neural tissue that contained more myelinated axons and potentially more complex internal structures. Consequently, brain networks in the treatment group likely maintained efficiency through functional redundancy and highly efficient neural circuits. Therefore, spinal cord DTI parameters, especially FA, serve as clinically pertinent biomarkers not only for local pathology [69] and offer critical insights into the mechanisms driving the reorganization of spinal cord–brain neural circuits.

The absence of B0 field-maps in scanning protocols and the omission of TopUp correction during data processing represent common and widely recognized issues in current rhesus monkey magnetic resonance imaging [70]. These factors are critical for mitigating geometric distortions arising from B0 field inhomogeneity [71]. To address this limitation, the present study utilized proton density-weighted images for geometric distortion correction [37,38]. Future work should integrate B0 field-maps or reverse-phase encoding images for TopUp correction to enhance data quality and statistical power.

### 4.4. Consistently Improved Clustering Coefficient over Time After Treatment

The Cp quantifies the brain’s capacity for functional segregation, with higher values typically reflecting more efficient local information processing [46,72]. In the present study, while no statistically significant longitudinal changes in Cp were detected within the functional network of the SCI-only group, there was an observable increasing trend over time. This trend could suggest a neuroplastic response to injury, where the brain augments local clustering as a compensatory mechanism for preserving information processing efficiency following the disruption of long-range connections [73]. Conversely, the SCN showed a significant longitudinal decrease in Cp post NT3 treatment. This reduction in structural network segregation implies that NT3 therapy mitigates tissue damage and promotes the establishment of cross-module integrative connections. Consequently, the network may reduce excessive local clustering, transitioning from a potentially inefficient compensatory architecture towards a more optimized and globally integrated state. This study also discovered that after SCI, the Cp of the SCN was proportional to the path length, reinforcing the propensity of brain regions to form denser local clusters post-SCI as a compensation for motor functions. The treatment notably altered this relationship pattern, promoting a more efficient topology. A similar relationship was observed between path length and λ, as well as between stride length and Cp. The corresponding interaction terms in these LMMs exhibited moderate-to-large effect sizes, collectively indicating a compensatory dependence on segregated network topology following SCI. It should be noted that although the selected time points (1, 2, 3, 6, and 12 months) cover key periods of post-injury repair, they may not capture neuroplastic fluctuations occurring between these intervals. Future studies using denser sampling schedules or chronic recording techniques will help clarify these finer-grained dynamics.

### 4.5. Altered Brain Network-Motor Function Relationship After SCI

The analyses revealed that the Eg, Eloc, and other metrics were strongly associated with path length, which was significantly changed after the treatment. The interaction between path length and these metrics at specific sparsity thresholds and their AUC values were consistent, suggesting that the findings were robust and generalizable. Eg is a parameter that quantifies the parallel information transmission capacity of a network [47]. In the SCI-only group, Eg was strongly and negatively associated with path length. This result aligns with Yang et al.’s report of an inverse relationship between the Eg of the structural network and motor scores in patients with complete SCI [19]. The negative correlation suggests that excessively high Eg in the SCN may reduce network flexibility and induce excessive integration, which eventually hinders motor recovery—a potential form of maladaptive neuroplasticity after SCI. In contrast, the treatment group showed the opposite trend of correlation, although it was not significant. NT3 treatment disrupted this pathological association, likely through its direct activation of TrkC receptors on neuronal surfaces within the impaired motor circuitry, which subsequently triggered intracellular pro-survival and pro-growth signaling pathways [74,75]. This treatment may enable ascending and descending neural signals to be relayed through newly formed synaptic connections [21]. These nascent, structurally redundant pathways restore flexibility and adaptability to the brain network, facilitating its reorganization into a more efficient configuration. Taken together, our results highlight that network parameters could serve as objective biomarkers for evaluating sensorimotor recovery potential after SCI and treatment.

The positive correlation found in our study between Eloc in the SCN and path length after SCI might suggest a compensatory strengthening of local connections to maintain motor functionality. Nevertheless, the treatment appears to have altered this relationship, causing it to trend negatively. This shift implies that the intervention encourages a more adaptable and streamlined network architecture, facilitating parallel information processing conducive to motor recovery.

### 4.6. Limitations

This study has some limitations. Firstly, the sample size is relatively small. Although we employed methods suitable for small samples in the linear mixed-effects model and adopted a conservative strategy by reporting effect sizes in the results, the limited sample size implies a risk of overinterpreting these correlations, suggesting the need for future research to incorporate a larger sample size to bolster the reliability of the results. Second, the current study primarily focuses on the global characteristics of brain networks, without specifically targeting individual brain regions or subnetworks. Furthermore, although the non-human primate model provides a valuable platform for investigating brain and spinal cord interactions, species differences necessitate caution when extrapolating these specific findings to clinical translation. Finally, the requirement for anesthesia in animal MRI is a double-edged sword: it is essential for subject immobilization [76,77] yet introduces a variable by influencing cerebral physiology [78]. However, studies have established that fMRI metrics under anesthesia show sufficient test–retest reliability for longitudinal investigations [79]. Accordingly, a consistent and rational anesthesia protocol was implemented to minimize its confounding effects on longitudinal comparisons, thereby enabling the investigation of large-scale brain networks.

## Data Availability

Upon reasonable request and with the consent of the study team, the study data are available from the corresponding author.

## Supplementary Materials

The following supporting information can be downloaded at: https://www.mdpi.com/article/10.3390/biomedicines13123124/s1. Table S1: Detailed statistical information on global metrics of functional network with significant within-group differences; Table S2: Detailed statistical information on global metrics of structural covariance network with significant within-group differences; Table S3: Detailed statistical information on global metrics of structural covariance network with significant between-group differences; Table S4: Detailed statistical information on spinal cord DTI parameters with significant within-group differences; Table S5: Detailed statistical information on spinal cord DTI parameters with significant between-group differences; Table S6: Detailed statistics from linear mixed-effects models testing the moderating role of regenerative therapy in the association between spinal cord DTI parameters and brain network metrics after SCI; Table S7: Detailed statistics from simple slopes analysis examining the association between spinal cord DTI parameters and brain network metrics after SCI, stratified by group; Table S8: Detailed statistics from paired comparison of slopes on spinal cord DTI parameters-brain network metrics associations between the SCI-only and treatment groups; Table S9: Variance explained and effect sizes from linear mixed effects models of spinal cord DTI parameters × group interactions on brain network metrics; Table S10: Detailed statistics from linear mixed-effects models testing the moderating role of regenerative therapy in the association between brain network metrics and locomotor parameters after SCI; Table S11: Detailed statistics from simple slopes analysis examining the association between brain network metrics and locomotor parameters after SCI, stratified by group; Table S12: Detailed statistics from paired comparison of slopes on brain network metrics-locomotor parameters associations between the SCI-only and treatment groups; Table S13: Variance explained and effect sizes from linear mixed effects models of brain network metric × group interactions on locomotor parameters; Figure S1: Experimental design, data processing, and statistical analysis pipeline; Figure S2: Between-group and within-group differences in small-world characteristics within functional network, including σ, γ and λ; Figure S3: The longitudinal trajectories of global metrics with significant changes with the duration of injury at sparsity range of 0.05–0.09 and 0.11–0.50; Figure S4: The longitudinal trajectories of global metrics that exhibit a downward or upward trend with the duration of injury at network sparsity thresholds of 0.10. References [80,81] are cited in the Supplementary Materials.
