# Supplementary material for: Longitudinal Changes in Brain Network Metrics and Their Correlations with Spinal Cord Diffusion Tensor Imaging Parameters Following Spinal Cord Injury and Regenerative Therapy
Source: Biomedicines. 2025 Dec 18;13(12):3124. doi: 10.3390/biomedicines13123124 (PMC12730989; doi:10.3390/biomedicines13123124)
Supplement: Supplementary file 1 [file biomedicines-13-03124-s001.zip › biomedicines-3952633-supplementary.pdf]

Supplementary material

Table S1. Detailed statistical information on global metrics of functional network with significant within-group differences.

σ, small-worldness; λ, normalized characteristic path length; γ , normalized clustering coefficient; Eloc, local efficiency; Cp, clustering coefficient; Lp, characteristic path length; Eg, glocal efficiency; AUC, area under the curve.

Metric	Group	Time point (months)	Normality p.value	Test Type	t-statistic	p.value	Mean difference	SD difference	Lower 95%CI	Upper 95%CI	Cohen's d
Lp_Sparsity_0.14	SCI-only	6	0.771135	Paired t-test	-3.18308	0.049976	-0.74953	0.470947	-1.49891	-0.00015	-1.59154
Lp_Sparsity_0.15	SCI-only	6	0.662685	Paired t-test	-3.22745	0.04831	-0.79165	0.490573	-1.57226	-0.01104	-1.61372
Lp_Sparsity_0.16	SCI-only	6	0.596232	Paired t-test	-3.2388	0.047895	-0.82043	0.506628	-1.62659	-0.01427	-1.6194
Lp_Sparsity_0.17	SCI-only	6	0.522232	Paired t-test	-3.24853	0.047543	-0.85406	0.525814	-1.69075	-0.01737	-1.62426
Lp_Sparsity_0.18	SCI-only	6	0.477475	Paired t-test	-3.24918	0.047519	-0.8819	0.542842	-1.74568	-0.01811	-1.62459
Lp_Sparsity_0.19	SCI-only	6	0.41793	Paired t-test	-3.25924	0.047159	-0.90625	0.556113	-1.79115	-0.02135	-1.62962
Lp_Sparsity_0.20	SCI-only	6	0.393976	Paired t-test	-3.25869	0.047178	-0.92207	0.565914	-1.82256	-0.02157	-1.62934
Lp_Sparsity_0.21	SCI-only	6	0.392385	Paired t-test	-3.24932	0.047514	-0.93691	0.576681	-1.85454	-0.01928	-1.62466
Lp_Sparsity_0.22	SCI-only	6	0.36914	Paired t-test	-3.25112	0.047449	-0.95097	0.585011	-1.88185	-0.02009	-1.62556
Lp_Sparsity_0.23	SCI-only	6	0.360296	Paired t-test	-3.24868	0.047537	-0.96226	0.592399	-1.9049	-0.01962	-1.62434
Lp_Sparsity_0.24	SCI-only	6	0.348114	Paired t-test	-3.2481	0.047558	-0.97323	0.599262	-1.92679	-0.01967	-1.62405
Lp_Sparsity_0.25	SCI-only	6	0.348833	Paired t-test	-3.24437	0.047693	-0.97992	0.604076	-1.94114	-0.0187	-1.62219
Lp_Sparsity_0.26	SCI-only	6	0.34753	Paired t-test	-3.24203	0.047777	-0.98568	0.608064	-1.95324	-0.01811	-1.62101
Lp_Sparsity_0.27	SCI-only	6	0.346472	Paired t-test	-3.24018	0.047844	-0.9902	0.611199	-1.96275	-0.01764	-1.62009
Lp_Sparsity_0.28	SCI-only	6	0.350637	Paired t-test	-3.23671	0.047971	-0.99366	0.613994	-1.97066	-0.01666	-1.61836
Lp_Sparsity_0.29	SCI-only	6	0.352462	Paired t-test	-3.23465	0.048046	-0.99634	0.616045	-1.97661	-0.01608	-1.61732
Lp_Sparsity_0.30	SCI-only	6	0.355231	Paired t-test	-3.23239	0.048128	-0.99856	0.617845	-1.98169	-0.01543	-1.6162
Lp_Sparsity_0.31	SCI-only	6	0.356355	Paired t-test	-3.23112	0.048175	-1.00023	0.61912	-1.98538	-0.01507	-1.61556
Lp_Sparsity_0.32	SCI-only	6	0.357202	Paired t-test	-3.23023	0.048208	-1.00134	0.619983	-1.98787	-0.01481	-1.61511

Metric	Group	Time point (months)	Normality p.value	Test Type	t-statistic	p.value	Mean difference	SD difference	Lower 95%CI	Upper 95%CI	Cohen's d
Lp_Sparsity_0.33	SCI-only	6	0.358332	Paired t-test	-3.22933	0.04824	-1.0022	0.620685	-1.98985	-0.01455	-1.61466
Lp_Sparsity_0.34	SCI-only	6	0.359345	Paired t-test	-3.22867	0.048265	-1.00266	0.621097	-1.99096	-0.01435	-1.61433
Lp_Sparsity_0.35	SCI-only	6	0.359855	Paired t-test	-3.22831	0.048278	-1.00294	0.621338	-1.99162	-0.01425	-1.61416
Lp_Sparsity_0.36	SCI-only	6	0.360287	Paired t-test	-3.22804	0.048288	-1.00313	0.621508	-1.99208	-0.01417	-1.61402
Lp_Sparsity_0.37	SCI-only	6	0.361016	Paired t-test	-3.22763	0.048303	-1.00331	0.621703	-1.99258	-0.01404	-1.61381
Lp_Sparsity_0.38	SCI-only	6	0.361215	Paired t-test	-3.22751	0.048307	-1.00337	0.621758	-1.99272	-0.01401	-1.61376
Lp_Sparsity_0.39	SCI-only	6	0.361505	Paired t-test	-3.22735	0.048313	-1.00343	0.621832	-1.99291	-0.01396	-1.61368
Lp_Sparsity_0.40	SCI-only	6	0.361635	Paired t-test	-3.22728	0.048316	-1.00346	0.621862	-1.99298	-0.01394	-1.61364
Lp_Sparsity_0.41	SCI-only	6	0.361733	Paired t-test	-3.22723	0.048318	-1.00348	0.621885	-1.99304	-0.01392	-1.61361
Lp_Sparsity_0.42	SCI-only	6	0.361752	Paired t-test	-3.22722	0.048318	-1.00349	0.621891	-1.99306	-0.01392	-1.61361
Lp_Sparsity_0.43	SCI-only	6	0.361769	Paired t-test	-3.22721	0.048318	-1.00349	0.621895	-1.99307	-0.01392	-1.6136
Lp_Sparsity_0.44	SCI-only	6	0.36178	Paired t-test	-3.2272	0.048319	-1.00349	0.621897	-1.99307	-0.01392	-1.6136
Lp_Sparsity_0.45	SCI-only	6	0.361799	Paired t-test	-3.22719	0.048319	-1.0035	0.621901	-1.99308	-0.01391	-1.6136
Lp_Sparsity_0.46	SCI-only	6	0.361807	Paired t-test	-3.22719	0.048319	-1.0035	0.621903	-1.99308	-0.01391	-1.61359
Lp_Sparsity_0.47	SCI-only	6	0.361809	Paired t-test	-3.22719	0.048319	-1.0035	0.621903	-1.99309	-0.01391	-1.61359
Lp_Sparsity_0.48	SCI-only	6	0.361812	Paired t-test	-3.22719	0.048319	-1.0035	0.621904	-1.99309	-0.01391	-1.61359
Lp_Sparsity_0.49	SCI-only	6	0.36182	Paired t-test	-3.22718	0.048319	-1.0035	0.621905	-1.99309	-0.01391	-1.61359
Lp_Sparsity_0.50	SCI-only	6	0.361827	Paired t-test	-3.22718	0.048319	-1.0035	0.621907	-1.99309	-0.01391	-1.61359
Lp_AUC	SCI-only	12	0.519659	Paired t-test	-3.46137	0.0406	-0.12878	0.074411	-0.24719	-0.01038	-1.73068
Cp_AUC	treatment	2	0.074322	Paired t-test	3.681773	0.034714	0.020528	0.011151	0.002784	0.038271	1.840886
Eloc_AUC	treatment	2	0.339781	Paired t-test	3.513624	0.039096	0.020064	0.01142	0.001891	0.038236	1.756812

Table S2. Detailed statistical information on global metrics of structural covariance network with significant within-group differences.

σ, small-worldness; λ, normalized characteristic path length; γ , normalized clustering coefficient; Eloc, local efficiency; Cp, clustering coefficient; Lp, characteristic path length; Eg, glocal efficiency; AUC, area under the curve.

Metric	Group	Time point (months)	Normality p.value	Test Type	t-statistic	p.value	Mean difference	SD difference	Lower 95%CI	Upper 95%CI	Cohen's d
λ_Sparsity_0.33	SCI-only	1	0.399557	Paired t-test	-3.62022	0.036242	-0.00338	0.001867	-0.00635	-0.00041	-1.81011
λ_Sparsity_0.34	SCI-only	1	0.408065	Paired t-test	-3.47755	0.040126	-0.00295	0.001695	-0.00564	-0.00025	-1.73878
λ_Sparsity_0.35	SCI-only	1	0.565709	Paired t-test	-3.45582	0.040764	-0.00265	0.001532	-0.00509	-0.00021	-1.72791
λ_Sparsity_0.36	SCI-only	1	0.501559	Paired t-test	-3.3004	0.04572	-0.00232	0.001405	-0.00456	-8.3E-05	-1.6502
λ_Sparsity_0.38	SCI-only	1	0.720619	Paired t-test	-3.24203	0.047777	-0.00181	0.001116	-0.00359	-3.3E-05	-1.62102
λ_Sparsity_0.40	SCI-only	1	0.973335	Paired t-test	-3.38019	0.043085	-0.00131	0.000776	-0.00255	-7.7E-05	-1.69009
λ_Sparsity_0.41	SCI-only	1	0.861448	Paired t-test	-3.301	0.045699	-0.00112	0.000677	-0.0022	-4E-05	-1.6505
λ_Sparsity_0.42	SCI-only	1	0.743175	Paired t-test	-3.39296	0.042682	-0.00108	0.000635	-0.00209	-6.7E-05	-1.69648
λ_Sparsity_0.44	SCI-only	1	0.497633	Paired t-test	-3.34067	0.044366	-0.00069	0.000411	-0.00134	-3.3E-05	-1.67033
λ_Sparsity_0.48	SCI-only	1	0.859987	Paired t-test	-3.30833	0.045449	-0.00039	0.000235	-0.00076	-1.5E-05	-1.65417
λ_Sparsity_0.50	SCI-only	1	0.975269	Paired t-test	-3.6673	0.035066	-0.00025	0.000134	-0.00046	-3.2E-05	-1.83365
Eg_Sparsity_0.33	SCI-only	1	0.504654	Paired t-test	3.392891	0.042684	0.00226	0.001332	0.00014	0.004381	1.696445
Eg_Sparsity_0.34	SCI-only	1	0.520621	Paired t-test	3.274126	0.046632	0.001992	0.001217	5.58E-05	0.003928	1.637063
Eg_Sparsity_0.35	SCI-only	1	0.657428	Paired t-test	3.283934	0.046289	0.001805	0.001099	5.58E-05	0.003555	1.641967
Eg_Sparsity_0.36	SCI-only	1	0.566506	Paired t-test	3.207227	0.04906	0.001602	0.000999	1.24E-05	0.003191	1.603614
Eg_Sparsity_0.40	SCI-only	1	0.95997	Paired t-test	3.287543	0.046163	0.000925	0.000563	2.96E-05	0.001821	1.643772
Eg_Sparsity_0.41	SCI-only	1	0.831758	Paired t-test	3.217703	0.048669	0.000795	0.000494	8.72E-06	0.001582	1.608851
Eg_Sparsity_0.42	SCI-only	1	0.711287	Paired t-test	3.335513	0.044536	0.000774	0.000464	3.55E-05	0.001513	1.667757
Eg_Sparsity_0.44	SCI-only	1	0.510299	Paired t-test	3.256143	0.047269	0.000498	0.000306	1.13E-05	0.000985	1.628071
Eg_Sparsity_0.48	SCI-only	1	0.868034	Paired t-test	3.202283	0.049246	0.000288	0.00018	1.78E-06	0.000574	1.601142
Eg_Sparsity_0.50	SCI-only	1	0.98145	Paired t-test	3.547221	0.038166	0.000183	0.000103	1.89E-05	0.000348	1.77361

Metric	Group	Time point (months)	Normality p.value	Test Type	t-statistic	p.value	Mean difference	SD difference	Lower 95%CI	Upper 95%CI	Cohen's d
Lp_Sparsity_0.33	SCI-only	1	0.517026	Paired t-test	-3.38784	0.042843	-0.00517	0.003055	-0.01004	-0.00031	-1.69392
Lp_Sparsity_0.34	SCI-only	1	0.530727	Paired t-test	-3.27024	0.046769	-0.00448	0.002743	-0.00885	-0.00012	-1.63512
Lp_Sparsity_0.35	SCI-only	1	0.663487	Paired t-test	-3.27952	0.046443	-0.004	0.002439	-0.00788	-0.00012	-1.63976
Lp_Sparsity_0.36	SCI-only	1	0.577075	Paired t-test	-3.20418	0.049174	-0.00349	0.002179	-0.00696	-2.4E-05	-1.60209
Lp_Sparsity_0.40	SCI-only	1	0.958419	Paired t-test	-3.28567	0.046228	-0.0019	0.001154	-0.00373	-6E-05	-1.64284
Lp_Sparsity_0.41	SCI-only	1	0.82914	Paired t-test	-3.21534	0.048757	-0.00161	0.000999	-0.00319	-1.6E-05	-1.60767
Lp_Sparsity_0.42	SCI-only	1	0.707462	Paired t-test	-3.3331	0.044616	-0.00154	0.000924	-0.00301	-7E-05	-1.66655
Lp_Sparsity_0.44	SCI-only	1	0.50738	Paired t-test	-3.25472	0.04732	-0.00096	0.000592	-0.0019	-2.1E-05	-1.62736
Lp_Sparsity_0.48	SCI-only	1	0.867233	Paired t-test	-3.20071	0.049305	-0.00053	0.000329	-0.00105	-3E-06	-1.60036
Lp_Sparsity_0.50	SCI-only	1	0.981463	Paired t-test	-3.54641	0.038188	-0.00033	0.000184	-0.00062	-3.3E-05	-1.77321
σ_Sparsity_0.05	SCI-only	3	0.47014	Paired t-test	4.030972	0.027448	0.408732	0.202796	0.086039	0.731426	2.015486
σ_Sparsity_0.06	SCI-only	3	0.756129	Paired t-test	3.363743	0.043612	0.324711	0.193065	0.017501	0.631921	1.681872
λ_Sparsity_0.11	SCI-only	3	0.05069	Paired t-test	-4.2572	0.023764	-0.0301	0.014142	-0.05261	-0.0076	-2.1286
λ_Sparsity_0.14	SCI-only	3	0.104196	Paired t-test	-4.4321	0.021344	-0.02527	0.011403	-0.04341	-0.00712	-2.21605
λ_Sparsity_0.15	SCI-only	3	0.232429	Paired t-test	-5.33641	0.012864	-0.02415	0.009053	-0.03856	-0.00975	-2.66821
λ_Sparsity_0.16	SCI-only	3	0.614882	Paired t-test	-5.49842	0.011839	-0.02172	0.007902	-0.0343	-0.00915	-2.74921
λ_Sparsity_0.17	SCI-only	3	0.772802	Paired t-test	-5.71976	0.010605	-0.02036	0.007118	-0.03168	-0.00903	-2.85988
λ_Sparsity_0.18	SCI-only	3	0.788658	Paired t-test	-7.10485	0.005737	-0.0184	0.00518	-0.02665	-0.01016	-3.55242
λ_Sparsity_0.19	SCI-only	3	0.47935	Paired t-test	-7.38027	0.005144	-0.01617	0.004382	-0.02315	-0.0092	-3.69014
λ_Sparsity_0.20	SCI-only	3	0.380786	Paired t-test	-6.87498	0.006303	-0.01463	0.004256	-0.0214	-0.00786	-3.43749
λ_Sparsity_0.21	SCI-only	3	0.673224	Paired t-test	-6.99466	0.005999	-0.0131	0.003745	-0.01906	-0.00714	-3.49733
λ_Sparsity_0.22	SCI-only	3	0.76717	Paired t-test	-6.52945	0.0073	-0.01169	0.00358	-0.01739	-0.00599	-3.26473
λ_Sparsity_0.23	SCI-only	3	0.339941	Paired t-test	-5.77421	0.010327	-0.01061	0.003674	-0.01645	-0.00476	-2.88711
λ_Sparsity_0.24	SCI-only	3	0.221658	Paired t-test	-5.32967	0.012909	-0.0096	0.003601	-0.01533	-0.00387	-2.66483

Metric	Group	Time point (months)	Normality p.value	Test Type	t-statistic	p.value	Mean difference	SD difference	Lower 95%CI	Upper 95%CI	Cohen's d
λ_Sparsity_0.25	SCI-only	3	0.426292	Paired t-test	-4.54039	0.020004	-0.00831	0.003659	-0.01413	-0.00248	-2.27019
λ_Sparsity_0.26	SCI-only	3	0.584735	Paired t-test	-3.94698	0.029002	-0.00729	0.003693	-0.01317	-0.00141	-1.97349
λ_Sparsity_0.27	SCI-only	3	0.392682	Paired t-test	-4.45484	0.021053	-0.00677	0.003038	-0.0116	-0.00193	-2.22742
λ_Sparsity_0.28	SCI-only	3	0.5966	Paired t-test	-4.08305	0.026538	-0.00602	0.002949	-0.01071	-0.00133	-2.04153
λ_Sparsity_0.29	SCI-only	3	0.628507	Paired t-test	-3.67975	0.034763	-0.00508	0.002762	-0.00948	-0.00069	-1.83987
λ_Sparsity_0.30	SCI-only	3	0.880782	Paired t-test	-3.25932	0.047156	-0.00422	0.002589	-0.00834	-1E-04	-1.62966
λ_Sparsity_0.39	SCI-only	3	0.153311	Paired t-test	-3.22605	0.048361	-0.00123	0.000761	-0.00244	-1.7E-05	-1.61303
λ_Sparsity_0.40	SCI-only	3	0.235737	Paired t-test	-3.58477	0.03716	-0.00107	0.000598	-0.00202	-0.00012	-1.79239
λ_Sparsity_0.41	SCI-only	3	0.052125	Paired t-test	-4.50325	0.020451	-0.00094	0.000418	-0.00161	-0.00028	-2.25163
λ_Sparsity_0.43	SCI-only	3	0.469432	Paired t-test	-5.04198	0.015043	-0.00075	0.000299	-0.00123	-0.00028	-2.52099
λ_Sparsity_0.44	SCI-only	3	0.484562	Paired t-test	-5.17968	0.013969	-0.00054	0.000209	-0.00087	-0.00021	-2.58984
λ_Sparsity_0.45	SCI-only	3	0.180724	Paired t-test	-4.89538	0.016309	-0.00046	0.000187	-0.00076	-0.00016	-2.44769
λ_Sparsity_0.46	SCI-only	3	0.902763	Paired t-test	-4.67117	0.018527	-0.00042	0.00018	-0.00071	-0.00013	-2.33559
λ_Sparsity_0.47	SCI-only	3	0.75799	Paired t-test	-3.63532	0.035859	-0.00037	0.000204	-0.0007	-4.6E-05	-1.81766
λ_AUC	SCI-only	3	0.13214	Paired t-test	-5.77582	0.010319	-0.00538	0.001864	-0.00835	-0.00242	-2.88791
Eg_Sparsity_0.05	SCI-only	3	0.446312	Paired t-test	3.615904	0.036352	0.018714	0.010351	0.002243	0.035185	1.807952
Eg_Sparsity_0.06	SCI-only	3	0.635449	Paired t-test	4.153552	0.025367	0.016437	0.007914	0.003843	0.02903	2.076776
Eg_Sparsity_0.07	SCI-only	3	0.641849	Paired t-test	5.511049	0.011764	0.017426	0.006324	0.007363	0.027488	2.755524
Eg_Sparsity_0.08	SCI-only	3	0.293572	Paired t-test	6.511626	0.007357	0.016913	0.005195	0.008647	0.025179	3.255813
Eg_Sparsity_0.09	SCI-only	3	0.589025	Paired t-test	7.609637	0.00471	0.017138	0.004504	0.00997	0.024305	3.804818
Eg_Sparsity_0.10	SCI-only	3	0.532601	Paired t-test	6.401588	0.007722	0.017104	0.005344	0.008601	0.025607	3.200794
Eg_Sparsity_0.11	SCI-only	3	0.476997	Paired t-test	6.881362	0.006286	0.015143	0.004401	0.00814	0.022147	3.440681
Eg_Sparsity_0.12	SCI-only	3	0.707399	Paired t-test	5.411831	0.012373	0.014047	0.005191	0.005787	0.022308	2.705915
Eg_Sparsity_0.13	SCI-only	3	0.885219	Paired t-test	5.518871	0.011717	0.013736	0.004978	0.005815	0.021656	2.759436

Metric	Group	Time point (months)	Normality p.value	Test Type	t-statistic	p.value	Mean difference	SD difference	Lower 95%CI	Upper 95%CI	Cohen's d
Eg_Sparsity_0.14	SCI-only	3	0.940962	Paired t-test	5.528164	0.011663	0.013769	0.004981	0.005843	0.021696	2.764082
Eg_Sparsity_0.15	SCI-only	3	0.95936	Paired t-test	6.209749	0.008416	0.012915	0.00416	0.006296	0.019534	3.104875
Eg_Sparsity_0.16	SCI-only	3	0.651795	Paired t-test	6.408559	0.007698	0.011732	0.003661	0.005906	0.017557	3.204279
Eg_Sparsity_0.17	SCI-only	3	0.475926	Paired t-test	6.653453	0.00692	0.011175	0.003359	0.00583	0.01652	3.326726
Eg_Sparsity_0.18	SCI-only	3	0.20711	Paired t-test	8.180798	0.003821	0.010136	0.002478	0.006193	0.014079	4.090399
Eg_Sparsity_0.19	SCI-only	3	0.963151	Paired t-test	9.521173	0.002457	0.009339	0.001962	0.006217	0.01246	4.760587
Eg_Sparsity_0.20	SCI-only	3	0.968163	Paired t-test	8.123057	0.0039	0.008475	0.002087	0.005155	0.011795	4.061528
Eg_Sparsity_0.21	SCI-only	3	0.820317	Paired t-test	7.990624	0.00409	0.00771	0.00193	0.004639	0.010781	3.995312
Eg_Sparsity_0.22	SCI-only	3	0.426566	Paired t-test	6.603421	0.00707	0.006905	0.002091	0.003577	0.010233	3.30171
Eg_Sparsity_0.23	SCI-only	3	0.920523	Paired t-test	5.471851	0.012	0.006317	0.002309	0.002643	0.009991	2.735926
Eg_Sparsity_0.24	SCI-only	3	0.807095	Paired t-test	5.18216	0.01395	0.005797	0.002237	0.002237	0.009357	2.59108
Eg_Sparsity_0.25	SCI-only	3	0.761515	Paired t-test	4.369479	0.022172	0.005121	0.002344	0.001391	0.008851	2.184739
Eg_Sparsity_0.26	SCI-only	3	0.68609	Paired t-test	3.756764	0.032963	0.004577	0.002437	0.0007	0.008455	1.878382
Eg_Sparsity_0.27	SCI-only	3	0.612478	Paired t-test	4.342483	0.022542	0.004303	0.001982	0.001149	0.007456	2.171242
Eg_Sparsity_0.28	SCI-only	3	0.507166	Paired t-test	3.85115	0.030916	0.003859	0.002004	0.00067	0.007049	1.925575
Eg_Sparsity_0.29	SCI-only	3	0.473354	Paired t-test	3.49753	0.039551	0.003313	0.001894	0.000298	0.006327	1.748765
Eg_Sparsity_0.40	SCI-only	3	0.131451	Paired t-test	3.378268	0.043146	0.000758	0.000449	4.4E-05	0.001473	1.689134
Eg_Sparsity_0.43	SCI-only	3	0.377554	Paired t-test	4.660042	0.018647	0.000548	0.000235	0.000174	0.000922	2.330021
Eg_Sparsity_0.44	SCI-only	3	0.330024	Paired t-test	4.868918	0.016552	0.000394	0.000162	0.000136	0.000651	2.434459
Eg_Sparsity_0.45	SCI-only	3	0.271605	Paired t-test	4.599739	0.019315	0.000334	0.000145	0.000103	0.000565	2.299869
Eg_Sparsity_0.46	SCI-only	3	0.94146	Paired t-test	4.613378	0.019162	0.000308	0.000134	9.57E-05	0.000521	2.306689
Eg_Sparsity_0.47	SCI-only	3	0.705459	Paired t-test	3.614313	0.036393	0.000274	0.000152	3.28E-05	0.000516	1.807156
Eg_AUC	SCI-only	3	0.969323	Paired t-test	9.030739	0.002867	0.002829	0.000627	0.001832	0.003826	4.515369
Cp_Sparsity_0.15	SCI-only	3	0.89979	Paired t-test	-4.61572	0.019135	-0.017	0.007366	-0.02872	-0.00528	-2.30786

Metric	Group	Time point (months)	Normality p.value	Test Type	t-statistic	p.value	Mean difference	SD difference	Lower 95%CI	Upper 95%CI	Cohen's d
Cp_Sparsity_0.16	SCI-only	3	0.697963	Paired t-test	-5.03893	0.015068	-0.01814	0.0072	-0.0296	-0.00668	-2.51946
Cp_Sparsity_0.17	SCI-only	3	0.186926	Paired t-test	-5.31749	0.012991	-0.01828	0.006877	-0.02923	-0.00734	-2.65874
Cp_Sparsity_0.18	SCI-only	3	0.117339	Paired t-test	-5.33881	0.012848	-0.01923	0.007204	-0.03069	-0.00777	-2.6694
Cp_Sparsity_0.19	SCI-only	3	0.417023	Paired t-test	-5.32627	0.012932	-0.02019	0.007581	-0.03225	-0.00813	-2.66313
Cp_Sparsity_0.20	SCI-only	3	0.377956	Paired t-test	-5.21828	0.013685	-0.01984	0.007605	-0.03194	-0.00774	-2.60914
Cp_Sparsity_0.21	SCI-only	3	0.537241	Paired t-test	-6.35888	0.00787	-0.02005	0.006307	-0.03009	-0.01002	-3.17944
Cp_Sparsity_0.22	SCI-only	3	0.60464	Paired t-test	-6.24748	0.008273	-0.02008	0.006428	-0.03031	-0.00985	-3.12374
Cp_Sparsity_0.23	SCI-only	3	0.314004	Paired t-test	-6.6256	0.007003	-0.02048	0.006182	-0.03032	-0.01064	-3.3128
Cp_Sparsity_0.24	SCI-only	3	0.085749	Paired t-test	-5.96536	0.009425	-0.01999	0.006702	-0.03065	-0.00932	-2.98268
Cp_Sparsity_0.25	SCI-only	3	0.220378	Paired t-test	-5.01537	0.015263	-0.01887	0.007523	-0.03084	-0.00689	-2.50768
Cp_Sparsity_0.26	SCI-only	3	0.544995	Paired t-test	-4.92526	0.01604	-0.01856	0.007535	-0.03055	-0.00657	-2.46263
Cp_Sparsity_0.27	SCI-only	3	0.600818	Paired t-test	-4.79501	0.017257	-0.01823	0.007602	-0.03032	-0.00613	-2.3975
Cp_Sparsity_0.28	SCI-only	3	0.596404	Paired t-test	-4.48261	0.020705	-0.01816	0.008104	-0.03106	-0.00527	-2.2413
Cp_Sparsity_0.29	SCI-only	3	0.372714	Paired t-test	-4.64788	0.01878	-0.01796	0.007729	-0.03026	-0.00566	-2.32394
Cp_Sparsity_0.30	SCI-only	3	0.389274	Paired t-test	-4.58819	0.019447	-0.01716	0.007482	-0.02907	-0.00526	-2.2941
Cp_Sparsity_0.31	SCI-only	3	0.61685	Paired t-test	-4.41081	0.021621	-0.01663	0.00754	-0.02863	-0.00463	-2.20541
Cp_Sparsity_0.32	SCI-only	3	0.761026	Paired t-test	-4.14094	0.025572	-0.01626	0.007854	-0.02876	-0.00376	-2.07047
Cp_Sparsity_0.33	SCI-only	3	0.830847	Paired t-test	-3.85869	0.03076	-0.01577	0.008173	-0.02877	-0.00276	-1.92935
Cp_Sparsity_0.34	SCI-only	3	0.77058	Paired t-test	-3.47184	0.040293	-0.01498	0.008628	-0.02871	-0.00125	-1.73592
Cp_Sparsity_0.35	SCI-only	3	0.817807	Paired t-test	-3.4029	0.042371	-0.01466	0.008618	-0.02838	-0.00095	-1.70145
Cp_AUC	SCI-only	3	0.869618	Paired t-test	-5.37512	0.012609	-0.00636	0.002367	-0.01013	-0.0026	-2.68756
Lp_Sparsity_0.05	SCI-only	3	0.42589	Paired t-test	-3.63819	0.035787	-0.12708	0.069861	-0.23825	-0.01592	-1.8191
Lp_Sparsity_0.06	SCI-only	3	0.604918	Paired t-test	-4.2459	0.023932	-0.09683	0.045611	-0.16941	-0.02425	-2.12295
Lp_Sparsity_0.07	SCI-only	3	0.698918	Paired t-test	-5.76787	0.010359	-0.09293	0.032224	-0.14421	-0.04166	-2.88394

Metric	Group	Time point (months)	Normality p.value	Test Type	t-statistic	p.value	Mean difference	SD difference	Lower 95%CI	Upper 95%CI	Cohen's d
Lp_Sparsity_0.08	SCI-only	3	0.294961	Paired t-test	-6.93441	0.00615	-0.08274	0.023864	-0.12071	-0.04477	-3.4672
Lp_Sparsity_0.09	SCI-only	3	0.593608	Paired t-test	-8.21001	0.003782	-0.07808	0.01902	-0.10834	-0.04781	-4.10501
Lp_Sparsity_0.10	SCI-only	3	0.535628	Paired t-test	-6.73275	0.00669	-0.07336	0.021793	-0.10804	-0.03869	-3.36637
Lp_Sparsity_0.11	SCI-only	3	0.498091	Paired t-test	-7.30583	0.005296	-0.0615	0.016836	-0.08829	-0.03471	-3.65292
Lp_Sparsity_0.12	SCI-only	3	0.716441	Paired t-test	-5.62477	0.011113	-0.05431	0.019312	-0.08504	-0.02358	-2.81238
Lp_Sparsity_0.13	SCI-only	3	0.882171	Paired t-test	-5.72728	0.010566	-0.05093	0.017785	-0.07923	-0.02263	-2.86364
Lp_Sparsity_0.14	SCI-only	3	0.935342	Paired t-test	-5.76236	0.010387	-0.04907	0.017032	-0.07617	-0.02197	-2.88118
Lp_Sparsity_0.15	SCI-only	3	0.971981	Paired t-test	-6.49326	0.007416	-0.04441	0.013679	-0.06618	-0.02264	-3.24663
Lp_Sparsity_0.16	SCI-only	3	0.646274	Paired t-test	-6.71331	0.006745	-0.03901	0.011623	-0.05751	-0.02052	-3.35665
Lp_Sparsity_0.17	SCI-only	3	0.44585	Paired t-test	-6.99662	0.005995	-0.03601	0.010293	-0.05239	-0.01963	-3.49831
Lp_Sparsity_0.18	SCI-only	3	0.182299	Paired t-test	-8.67327	0.003225	-0.03175	0.007321	-0.0434	-0.0201	-4.33664
Lp_Sparsity_0.19	SCI-only	3	0.885577	Paired t-test	-9.85865	0.002219	-0.0285	0.005781	-0.0377	-0.0193	-4.92932
Lp_Sparsity_0.20	SCI-only	3	0.997714	Paired t-test	-8.241	0.003741	-0.02523	0.006122	-0.03497	-0.01549	-4.1205
Lp_Sparsity_0.21	SCI-only	3	0.699945	Paired t-test	-8.00622	0.004067	-0.02239	0.005594	-0.0313	-0.01349	-4.00311
Lp_Sparsity_0.22	SCI-only	3	0.405848	Paired t-test	-6.60349	0.00707	-0.01959	0.005934	-0.02904	-0.01015	-3.30175
Lp_Sparsity_0.23	SCI-only	3	0.883924	Paired t-test	-5.48057	0.011947	-0.01753	0.006395	-0.0277	-0.00735	-2.74029
Lp_Sparsity_0.24	SCI-only	3	0.771485	Paired t-test	-5.18996	0.013892	-0.01572	0.00606	-0.02537	-0.00608	-2.59498
Lp_Sparsity_0.25	SCI-only	3	0.70831	Paired t-test	-4.37249	0.022131	-0.01361	0.006226	-0.02352	-0.0037	-2.18624
Lp_Sparsity_0.26	SCI-only	3	0.635111	Paired t-test	-3.76024	0.032885	-0.01192	0.006341	-0.02201	-0.00183	-1.88012
Lp_Sparsity_0.27	SCI-only	3	0.567851	Paired t-test	-4.34095	0.022563	-0.01098	0.00506	-0.01903	-0.00293	-2.17048
Lp_Sparsity_0.28	SCI-only	3	0.462275	Paired t-test	-3.85	0.03094	-0.00967	0.005021	-0.01765	-0.00168	-1.925
Lp_Sparsity_0.29	SCI-only	3	0.434402	Paired t-test	-3.49812	0.039535	-0.00814	0.004655	-0.01555	-0.00073	-1.74906
Lp_Sparsity_0.40	SCI-only	3	0.125637	Paired t-test	-3.37947	0.043108	-0.00155	0.00092	-0.00302	-9.1E-05	-1.68974
Lp_Sparsity_0.43	SCI-only	3	0.379079	Paired t-test	-4.6557	0.018694	-0.00107	0.000462	-0.00181	-0.00034	-2.32785

Metric	Group	Time point (months)	Normality p.value	Test Type	t-statistic	p.value	Mean difference	SD difference	Lower 95%CI	Upper 95%CI	Cohen's d
Lp_Sparsity_0.44	SCI-only	3	0.323056	Paired t-test	-4.86961	0.016546	-0.00076	0.000312	-0.00126	-0.00026	-2.43481
Lp_Sparsity_0.45	SCI-only	3	0.277012	Paired t-test	-4.59948	0.019318	-0.00064	0.000277	-0.00108	-0.0002	-2.29974
Lp_Sparsity_0.46	SCI-only	3	0.943314	Paired t-test	-4.61072	0.019191	-0.00058	0.000251	-0.00098	-0.00018	-2.30536
Lp_Sparsity_0.47	SCI-only	3	0.70656	Paired t-test	-3.6122	0.036447	-0.00051	0.000282	-0.00096	-6.1E-05	-1.8061
Lp_AUC	SCI-only	3	0.754768	Paired t-test	-8.05054	0.004003	-0.01082	0.002689	-0.0151	-0.00655	-4.02527
Eloc_Sparsity_0.15	SCI-only	3	0.649146	Paired t-test	-4.42235	0.02147	-0.00827	0.003741	-0.01422	-0.00232	-2.21117
Eloc_Sparsity_0.16	SCI-only	3	0.478686	Paired t-test	-5.00109	0.015383	-0.00889	0.003557	-0.01455	-0.00323	-2.50055
Eloc_Sparsity_0.17	SCI-only	3	0.312536	Paired t-test	-5.36566	0.012671	-0.00893	0.003328	-0.01422	-0.00363	-2.68283
Eloc_Sparsity_0.18	SCI-only	3	0.172748	Paired t-test	-5.51927	0.011715	-0.00956	0.003465	-0.01508	-0.00405	-2.75963
Eloc_Sparsity_0.19	SCI-only	3	0.407851	Paired t-test	-5.56063	0.011474	-0.01009	0.003629	-0.01586	-0.00432	-2.78032
Eloc_Sparsity_0.20	SCI-only	3	0.338863	Paired t-test	-5.44472	0.012167	-0.00992	0.003644	-0.01572	-0.00412	-2.72236
Eloc_Sparsity_0.21	SCI-only	3	0.424815	Paired t-test	-7.93567	0.004173	-0.01031	0.002598	-0.01444	-0.00617	-3.96784
Eloc_Sparsity_0.22	SCI-only	3	0.401115	Paired t-test	-7.733	0.004497	-0.01035	0.002677	-0.01461	-0.00609	-3.8665
Eloc_Sparsity_0.23	SCI-only	3	0.258572	Paired t-test	-7.98872	0.004093	-0.01057	0.002646	-0.01478	-0.00636	-3.99436
Eloc_Sparsity_0.25	SCI-only	3	0.269812	Paired t-test	-5.01629	0.015256	-0.00931	0.003713	-0.01522	-0.0034	-2.50814
Eloc_Sparsity_0.26	SCI-only	3	0.591167	Paired t-test	-4.91119	0.016166	-0.00917	0.003736	-0.01512	-0.00323	-2.45559
Eloc_Sparsity_0.27	SCI-only	3	0.648427	Paired t-test	-4.77708	0.017434	-0.00902	0.003778	-0.01503	-0.00301	-2.38854
Eloc_Sparsity_0.28	SCI-only	3	0.657561	Paired t-test	-4.46307	0.020949	-0.009	0.004035	-0.01542	-0.00258	-2.23154
Eloc_Sparsity_0.29	SCI-only	3	0.426942	Paired t-test	-4.63874	0.01888	-0.00891	0.003843	-0.01503	-0.0028	-2.31937
Eloc_Sparsity_0.30	SCI-only	3	0.341704	Paired t-test	-4.59455	0.019374	-0.00851	0.003706	-0.01441	-0.00262	-2.29728
Eloc_Sparsity_0.31	SCI-only	3	0.57583	Paired t-test	-4.41824	0.021524	-0.00825	0.003737	-0.0142	-0.00231	-2.20912
Eloc_Sparsity_0.32	SCI-only	3	0.732278	Paired t-test	-4.14773	0.025461	-0.00808	0.003898	-0.01429	-0.00188	-2.07386
Eloc_Sparsity_0.33	SCI-only	3	0.813572	Paired t-test	-3.86728	0.030583	-0.00785	0.004058	-0.01431	-0.00139	-1.93364
Eloc_Sparsity_0.34	SCI-only	3	0.756321	Paired t-test	-3.47846	0.0401	-0.00746	0.004288	-0.01428	-0.00063	-1.73923

Metric	Group	Time point (months)	Normality p.value	Test Type	t-statistic	p.value	Mean difference	SD difference	Lower 95%CI	Upper 95%CI	Cohen's d
Eloc_Sparsity_0.35	SCI-only	3	0.805249	Paired t-test	-3.40758	0.042226	-0.0073	0.004286	-0.01412	-0.00048	-1.70379
λ_Sparsity_0.49	SCI-only	6	0.401836	Paired t-test	-3.21021	0.048948	-0.0002	0.000127	-0.00041	-1.8E-06	-1.60511
Cp_Sparsity_0.07	treatment	2	0.966566	Paired t-test	4.068644	0.026786	0.003782	0.001859	0.000824	0.00674	2.034322
λ_Sparsity_0.05	treatment	6	0.352716	Paired t-test	-4.63101	0.018965	-0.06916	0.029866	-0.11668	-0.02163	-2.31551
λ_Sparsity_0.06	treatment	6	0.739243	Paired t-test	-3.88815	0.030159	-0.05585	0.028728	-0.10156	-0.01014	-1.94407
λ_Sparsity_0.07	treatment	6	0.98543	Paired t-test	-4.01107	0.027806	-0.04193	0.020909	-0.07521	-0.00866	-2.00554
λ_Sparsity_0.08	treatment	6	0.997217	Paired t-test	-4.09029	0.026415	-0.03276	0.016019	-0.05825	-0.00727	-2.04515
λ_Sparsity_0.09	treatment	6	0.629304	Paired t-test	-4.5901	0.019425	-0.02599	0.011325	-0.04401	-0.00797	-2.29505
λ_Sparsity_0.10	treatment	6	0.365388	Paired t-test	-3.79649	0.032081	-0.02144	0.011293	-0.03941	-0.00347	-1.89825
λ_Sparsity_0.11	treatment	6	0.848614	Paired t-test	-3.68842	0.034555	-0.01849	0.010024	-0.03444	-0.00254	-1.84421
λ_Sparsity_0.13	treatment	6	0.726501	Paired t-test	-3.22085	0.048553	-0.01352	0.008395	-0.02688	-0.00016	-1.61043
λ_AUC	treatment	6	0.336571	Paired t-test	-3.19241	0.049619	-0.0032	0.002006	-0.00639	-1E-05	-1.5962
Eg_Sparsity_0.05	treatment	6	0.863929	Paired t-test	5.120861	0.014415	0.028619	0.011178	0.010833	0.046405	2.56043
Eg_Sparsity_0.06	treatment	6	0.68956	Paired t-test	4.287683	0.023318	0.02464	0.011494	0.006352	0.042929	2.143842
Eg_Sparsity_0.07	treatment	6	0.126563	Paired t-test	4.490203	0.020611	0.0215	0.009576	0.006262	0.036738	2.245102
Eg_Sparsity_0.08	treatment	6	0.306709	Paired t-test	3.827404	0.031416	0.018655	0.009748	0.003144	0.034167	1.913702
Eg_Sparsity_0.09	treatment	6	0.494521	Paired t-test	3.683263	0.034678	0.015244	0.008277	0.002073	0.028415	1.841631
Eg_Sparsity_0.10	treatment	6	0.683966	Paired t-test	3.433804	0.041423	0.012627	0.007354	0.000924	0.024329	1.716902
Lp_Sparsity_0.05	treatment	6	0.798548	Paired t-test	-4.73252	0.017883	-0.19579	0.08274	-0.32744	-0.06413	-2.36626
Lp_Sparsity_0.06	treatment	6	0.670646	Paired t-test	-4.07947	0.026599	-0.1478	0.072462	-0.26311	-0.0325	-2.03974
Lp_Sparsity_0.07	treatment	6	0.110992	Paired t-test	-4.24421	0.023958	-0.11584	0.054589	-0.20271	-0.02898	-2.1221
Lp_Sparsity_0.08	treatment	6	0.287979	Paired t-test	-3.69327	0.034438	-0.09207	0.04986	-0.17141	-0.01273	-1.84663
Lp_Sparsity_0.09	treatment	6	0.484324	Paired t-test	-3.5772	0.037361	-0.06988	0.03907	-0.13205	-0.00771	-1.7886
Lp_Sparsity_0.10	treatment	6	0.684555	Paired t-test	-3.36156	0.043683	-0.0544	0.032368	-0.10591	-0.0029	-1.68078

Metric	Group	Time point (months)	Normality p.value	Test Type	t-statistic	p.value	Mean difference	SD difference	Lower 95%CI	Upper 95%CI	Cohen's d
λ_Sparsity_0.29	treatment	12	0.241494	Paired t-test	3.279349	0.046449	0.002008	0.001225	5.93E-05	0.003957	1.639675
λ_Sparsity_0.30	treatment	12	0.584402	Paired t-test	4.545075	0.019948	0.001806	0.000795	0.000542	0.003071	2.272537
λ_Sparsity_0.31	treatment	12	0.658053	Paired t-test	3.626741	0.036076	0.00188	0.001037	0.00023	0.00353	1.813371
λ_Sparsity_0.32	treatment	12	0.730069	Paired t-test	3.661591	0.035206	0.001653	0.000903	0.000216	0.00309	1.830796
λ_Sparsity_0.35	treatment	12	0.825056	Paired t-test	5.730294	0.01055	0.001387	0.000484	0.000617	0.002157	2.865147
λ_Sparsity_0.36	treatment	12	0.893231	Paired t-test	5.074039	0.014784	0.001398	0.000551	0.000521	0.002275	2.537019
λ_Sparsity_0.37	treatment	12	0.365208	Paired t-test	3.788691	0.032252	0.001282	0.000677	0.000205	0.002359	1.894346
λ_Sparsity_0.38	treatment	12	0.695928	Paired t-test	4.049429	0.027121	0.001426	0.000704	0.000305	0.002546	2.024715
λ_Sparsity_0.39	treatment	12	0.942629	Paired t-test	3.819267	0.031589	0.001232	0.000645	0.000205	0.002259	1.909634
λ_Sparsity_0.40	treatment	12	0.671443	Paired t-test	4.065521	0.02684	0.001134	0.000558	0.000246	0.002022	2.032761
λ_Sparsity_0.41	treatment	12	0.8263	Paired t-test	5.853551	0.00994	0.001253	0.000428	0.000572	0.001935	2.926775
λ_Sparsity_0.42	treatment	12	0.931775	Paired t-test	5.465232	0.01204	0.001155	0.000423	0.000482	0.001827	2.732616
λ_Sparsity_0.43	treatment	12	0.974819	Paired t-test	4.893536	0.016326	0.001031	0.000421	0.00036	0.001701	2.446768
λ_Sparsity_0.44	treatment	12	0.391054	Paired t-test	4.152364	0.025386	0.000966	0.000465	0.000226	0.001706	2.076182
λ_Sparsity_0.45	treatment	12	0.960122	Paired t-test	5.392705	0.012495	0.000834	0.000309	0.000342	0.001326	2.696352
λ_Sparsity_0.46	treatment	12	0.310767	Paired t-test	4.452285	0.021085	0.000677	0.000304	0.000193	0.00116	2.226143
λ_Sparsity_0.48	treatment	12	0.219722	Paired t-test	3.921067	0.029504	0.000536	0.000273	0.000101	0.000971	1.960534
λ_Sparsity_0.49	treatment	12	0.110996	Paired t-test	3.235353	0.04802	0.000491	0.000303	8.02E-06	0.000973	1.617676
λ_Sparsity_0.50	treatment	12	0.290647	Paired t-test	3.532331	0.038575	0.000451	0.000255	4.47E-05	0.000857	1.766165
Eg_Sparsity_0.22	treatment	12	0.599817	Paired t-test	-3.43757	0.041309	-0.00238	0.001387	-0.00459	-0.00018	-1.71878
Eg_Sparsity_0.23	treatment	12	0.412713	Paired t-test	-5.4149	0.012354	-0.00256	0.000944	-0.00406	-0.00105	-2.70745
Eg_Sparsity_0.24	treatment	12	0.35061	Paired t-test	-5.42468	0.012292	-0.00242	0.000892	-0.00384	-0.001	-2.71234
Eg_Sparsity_0.25	treatment	12	0.286983	Paired t-test	-5.87185	0.009853	-0.00253	0.000861	-0.0039	-0.00116	-2.93593
Eg_Sparsity_0.26	treatment	12	0.287332	Paired t-test	-8.88477	0.003007	-0.00237	0.000534	-0.00322	-0.00152	-4.44238

Metric	Group	Time point (months)	Normality p.value	Test Type	t-statistic	p.value	Mean difference	SD difference	Lower 95%CI	Upper 95%CI	Cohen's d
Eg_Sparsity_0.27	treatment	12	0.80814	Paired t-test	-13.7279	0.000836	-0.00227	0.000331	-0.0028	-0.00175	-6.86394
Eg_Sparsity_0.28	treatment	12	0.060876	Paired t-test	-11.5161	0.001406	-0.00225	0.00039	-0.00287	-0.00163	-5.75806
Eg_Sparsity_0.29	treatment	12	0.267836	Paired t-test	-14.5652	0.000702	-0.00226	0.00031	-0.00275	-0.00176	-7.2826
Eg_Sparsity_0.30	treatment	12	0.739379	Paired t-test	-7.3716	0.005161	-0.00196	0.000532	-0.00281	-0.00111	-3.6858
Eg_Sparsity_0.31	treatment	12	0.053457	Paired t-test	-7.00018	0.005986	-0.00199	0.000569	-0.0029	-0.00109	-3.50009
Eg_Sparsity_0.32	treatment	12	0.443017	Paired t-test	-6.49368	0.007415	-0.00182	0.00056	-0.00271	-0.00093	-3.24684
Eg_Sparsity_0.33	treatment	12	0.544266	Paired t-test	-6.50499	0.007378	-0.00169	0.00052	-0.00252	-0.00086	-3.25249
Eg_Sparsity_0.34	treatment	12	0.848298	Paired t-test	-5.70391	0.010687	-0.00158	0.000555	-0.00247	-0.0007	-2.85195
Eg_Sparsity_0.35	treatment	12	0.276775	Paired t-test	-4.37589	0.022085	-0.0016	0.00073	-0.00276	-0.00044	-2.18795
Eg_Sparsity_0.36	treatment	12	0.506441	Paired t-test	-4.48733	0.020646	-0.00161	0.000719	-0.00276	-0.00047	-2.24366
Eg_Sparsity_0.37	treatment	12	0.790218	Paired t-test	-4.64412	0.018821	-0.00154	0.000664	-0.0026	-0.00049	-2.32206
Eg_Sparsity_0.38	treatment	12	0.926097	Paired t-test	-4.30259	0.023103	-0.00162	0.000751	-0.00281	-0.00042	-2.1513
Eg_Sparsity_0.39	treatment	12	0.643655	Paired t-test	-4.10725	0.026129	-0.00146	0.000713	-0.0026	-0.00033	-2.05362
Eg_Sparsity_0.40	treatment	12	0.581166	Paired t-test	-3.44364	0.041127	-0.00139	0.000809	-0.00268	-0.00011	-1.72182
Eg_Sparsity_0.41	treatment	12	0.298088	Paired t-test	-5.64935	0.010978	-0.00103	0.000364	-0.00161	-0.00045	-2.82468
Eg_Sparsity_0.42	treatment	12	0.265181	Paired t-test	-5.06409	0.014864	-0.00095	0.000375	-0.00154	-0.00035	-2.53205
Eg_Sparsity_0.43	treatment	12	0.327325	Paired t-test	-4.37119	0.022149	-0.00085	0.00039	-0.00147	-0.00023	-2.1856
Eg_Sparsity_0.44	treatment	12	0.531808	Paired t-test	-4.24244	0.023984	-0.00081	0.00038	-0.00141	-0.0002	-2.12122
Eg_Sparsity_0.45	treatment	12	0.481241	Paired t-test	-4.56935	0.019664	-0.0007	0.000307	-0.00119	-0.00021	-2.28467
Eg_Sparsity_0.46	treatment	12	0.385036	Paired t-test	-4.46845	0.020881	-0.00055	0.000247	-0.00094	-0.00016	-2.23423
Eg_Sparsity_0.47	treatment	12	0.398501	Paired t-test	-4.52919	0.020137	-0.00049	0.000218	-0.00084	-0.00015	-2.2646
Eg_Sparsity_0.48	treatment	12	0.385395	Paired t-test	-4.04196	0.027253	-0.00044	0.00022	-0.00079	-9.4E-05	-2.02098
Eg_Sparsity_0.49	treatment	12	0.380741	Paired t-test	-3.47013	0.040343	-0.00041	0.000237	-0.00079	-3.4E-05	-1.73506
Eg_Sparsity_0.50	treatment	12	0.453885	Paired t-test	-3.66716	0.035069	-0.00038	0.000208	-0.00071	-5E-05	-1.83358

Metric	Group	Time point (months)	Normality p.value	Test Type	t-statistic	p.value	Mean difference	SD difference	Lower 95%CI	Upper 95%CI	Cohen's d
Lp_Sparsity_0.22	treatment	12	0.617062	Paired t-test	3.46356	0.040535	0.006778	0.003914	0.00055	0.013005	1.73178
Lp_Sparsity_0.23	treatment	12	0.313698	Paired t-test	5.510192	0.011769	0.007111	0.002581	0.003004	0.011218	2.755096
Lp_Sparsity_0.24	treatment	12	0.458544	Paired t-test	5.397281	0.012466	0.00659	0.002442	0.002704	0.010476	2.69864
Lp_Sparsity_0.25	treatment	12	0.394245	Paired t-test	5.853944	0.009938	0.006749	0.002306	0.00308	0.010417	2.926972
Lp_Sparsity_0.26	treatment	12	0.439673	Paired t-test	8.902717	0.002989	0.006198	0.001392	0.003983	0.008414	4.451358
Lp_Sparsity_0.27	treatment	12	0.936776	Paired t-test	13.61645	0.000857	0.005827	0.000856	0.004465	0.007189	6.808227
Lp_Sparsity_0.29	treatment	12	0.301199	Paired t-test	13.74912	0.000833	0.005567	0.00081	0.004279	0.006856	6.87456
Lp_Sparsity_0.30	treatment	12	0.710808	Paired t-test	7.204208	0.005513	0.004753	0.00132	0.002653	0.006853	3.602104
Lp_Sparsity_0.32	treatment	12	0.432589	Paired t-test	6.373781	0.007818	0.004251	0.001334	0.002129	0.006374	3.186891
Lp_Sparsity_0.33	treatment	12	0.5275	Paired t-test	6.395472	0.007743	0.003892	0.001217	0.001955	0.005828	3.197736
Lp_Sparsity_0.34	treatment	12	0.838266	Paired t-test	5.630875	0.011079	0.003579	0.001271	0.001556	0.005603	2.815437
Lp_Sparsity_0.35	treatment	12	0.286539	Paired t-test	4.342217	0.022546	0.003554	0.001637	0.000949	0.006159	2.171108
Lp_Sparsity_0.36	treatment	12	0.512546	Paired t-test	4.45171	0.021093	0.003533	0.001587	0.001007	0.006058	2.225855
Lp_Sparsity_0.37	treatment	12	0.794181	Paired t-test	4.608686	0.019214	0.003323	0.001442	0.001028	0.005617	2.304343
Lp_Sparsity_0.38	treatment	12	0.925692	Paired t-test	4.273871	0.023519	0.003424	0.001602	0.000874	0.005974	2.136936
Lp_Sparsity_0.39	treatment	12	0.643354	Paired t-test	4.082671	0.026545	0.003054	0.001496	0.000673	0.005434	2.041335
Lp_Sparsity_0.40	treatment	12	0.580921	Paired t-test	3.427863	0.041603	0.002862	0.00167	0.000205	0.005519	1.713932
Lp_Sparsity_0.41	treatment	12	0.302394	Paired t-test	5.626342	0.011104	0.002079	0.000739	0.000903	0.003254	2.813171
Lp_Sparsity_0.42	treatment	12	0.264918	Paired t-test	5.045853	0.015012	0.00189	0.000749	0.000698	0.003082	2.522926
Lp_Sparsity_0.43	treatment	12	0.326265	Paired t-test	4.358382	0.022323	0.001675	0.000769	0.000452	0.002898	2.179191
Lp_Sparsity_0.44	treatment	12	0.530696	Paired t-test	4.232075	0.02414	0.001559	0.000737	0.000387	0.002731	2.116037
Lp_Sparsity_0.45	treatment	12	0.481334	Paired t-test	4.558988	0.019785	0.00134	0.000588	0.000405	0.002275	2.279494
Lp_Sparsity_0.46	treatment	12	0.382851	Paired t-test	4.460475	0.020982	0.001036	0.000465	0.000297	0.001776	2.230237
Lp_Sparsity_0.47	treatment	12	0.397426	Paired t-test	4.522671	0.020215	0.000915	0.000405	0.000271	0.001559	2.261335

Metric	Group	Time point (months)	Normality p.value	Test Type	t-statistic	p.value	Mean difference	SD difference	Lower 95%CI	Upper 95%CI	Cohen’s d
Lp_Sparsity_0.48	treatment	12	0.383961	Paired t-test	4.037648	0.027329	0.000813	0.000403	0.000172	0.001454	2.018824
Lp_Sparsity_0.49	treatment	12	0.380006	Paired t-test	3.467683	0.040414	0.000743	0.000429	6.11E-05	0.001425	1.733841
Lp_Sparsity_0.50	treatment	12	0.453601	Paired t-test	3.664635	0.035131	0.000678	0.00037	8.92E-05	0.001267	1.832317
Eloc_Sparsity_0.08	treatment	12	0.464224	Paired t-test	-4.02134	0.027621	-0.00963	0.00479	-0.01725	-0.00201	-2.01067
Eloc_Sparsity_0.10	treatment	12	0.988356	Paired t-test	-5.45159	0.012124	-0.01015	0.003725	-0.01608	-0.00423	-2.72579
Eloc_Sparsity_0.11	treatment	12	0.715531	Paired t-test	-3.5793	0.037305	-0.00993	0.005551	-0.01877	-0.0011	-1.78965
Eloc_Sparsity_0.12	treatment	12	0.995517	Paired t-test	-3.30952	0.045408	-0.01133	0.006846	-0.02222	-0.00043	-1.65476

Table S3. Detailed statistical information on global metrics of structural covariance network with significant between-group differences.

σ, small-worldness; λ, normalized characteristic path length; γ , normalized clustering coefficient; Eloc, local efficiency; Cp, clustering coefficient; Lp, characteristic path length; Eg, glocal efficiency; AUC, area under the curve.

Metric	Mean SCI-only	Mean treatment	Mean difference	t statistic	df	p.value	Cohens'd	Lower 95%CI	Upper 95%CI	Test	Normality Both	Variance p.value
σ_Sparsity_0.05	2.871535	3.618247	-0.7467118	-3.22815413	6	0.017952	-2.28265	-1.31271	-0.18071	Student's t-test (equal variances)	Yes	0.832348
σ_Sparsity_0.06	2.661983	3.234011	-0.5720284	-2.95403502	6	0.025479	-2.08882	-1.04586	-0.0982	Student's t-test (equal variances)	Yes	0.749075
σ_Sparsity_0.07	2.503529	2.999546	-0.4960177	-2.99190992	6	0.02426	-2.1156	-0.90168	-0.09035	Student's t-test (equal variances)	Yes	0.587763
σ_Sparsity_0.08	2.369804	2.789644	-0.4198407	-2.60018711	6	0.040652	-1.83861	-0.81493	-0.02475	Student's t-test (equal variances)	Yes	0.460565
λ_Sparsity_0.36	1.002524	1.005458	-0.0029347	-2.45492749	6	0.049459	-1.7359	-0.00586	-9.6E-06	Student's t-test (equal variances)	Yes	0.680999
λ_Sparsity_0.37	1.002105	1.004762	-0.0026568	-2.57927079	6	0.04181	-1.82382	-0.00518	-0.00014	Student's t-test (equal variances)	Yes	0.683881
λ_Sparsity_0.38	1.001769	1.004253	-0.002484	-2.72528924	6	0.0344	-1.92707	-0.00471	-0.00025	Student's t-test (equal variances)	Yes	0.636554
λ_Sparsity_0.39	1.001537	1.003619	-0.0020821	-2.61020738	6	0.040109	-1.8457	-0.00403	-0.00013	Student's t-test (equal variances)	Yes	0.749576
λ_Sparsity_0.40	1.001212	1.00317	-0.0019578	-2.78402922	6	0.031827	-1.96861	-0.00368	-0.00024	Student's t-test (equal variances)	Yes	0.545101
λ_Sparsity_0.41	1.001074	1.002953	-0.0018797	-2.74384784	6	0.033564	-1.94019	-0.00356	-0.0002	Student's t-test (equal variances)	Yes	0.500145

Metric	Mean SCI-only	Mean treatment	Mean difference	t statistic	df	p.value	Cohens'd	Lower 95%CI	Upper 95%CI	Test	Normality Both	Variance p.value
λ_Sparsity_0.42	1.000919	1.00252	-0.0016018	-2.6586336	6	0.037593	-1.87994	-0.00308	-0.00013	Student's t-test (equal variances)	Yes	0.497879
λ_Sparsity_0.43	1.000703	1.002172	-0.0014696	-2.74524386	6	0.033502	-1.94118	-0.00278	-0.00016	Student's t-test (equal variances)	Yes	0.22423
λ_Sparsity_0.44	1.000584	1.001854	-0.0012706	-2.72707361	6	0.034319	-1.92833	-0.00241	-0.00013	Student's t-test (equal variances)	Yes	0.175683
λ_Sparsity_0.45	1.000491	1.001567	-0.0010762	-2.87961823	6	0.028072	-2.0362	-0.00199	-0.00016	Student's t-test (equal variances)	Yes	0.205758
λ_Sparsity_0.46	1.000354	1.001275	-0.0009208	-2.92060355	6	0.02661	-2.06518	-0.00169	-0.00015	Student's t-test (equal variances)	Yes	0.159058
λ_Sparsity_0.47	1.000232	1.001082	-0.00085	-3.40403139	6	0.014424	-2.40701	-0.00146	-0.00024	Student's t-test (equal variances)	Yes	0.065853
λ_Sparsity_0.48	1.000157	1.000923	-0.0007664	-3.41820701	3.299437	0.036208	-2.41704	-0.00144	-8.8E-05	Welch's t-test (unequal variances)	Yes	0.034804
λ_Sparsity_0.49	1.000121	1.000792	-0.0006709	-3.31169088	3.22467	0.040778	-2.34172	-0.00129	-5.1E-05	Welch's t-test (unequal variances)	Yes	0.023075
λ_Sparsity_0.50	1.00008	1.00069	-0.0006098	-3.37230652	3.137719	0.040513	-2.38458	-0.00117	-4.8E-05	Welch's t-test (unequal variances)	Yes	0.011344
Eg_Sparsity_0.37	0.683447	0.681032	0.00241517	2.490836969	6	0.047109	1.761288	4.26E-05	0.004788	Student's t-test (equal variances)	Yes	0.366225

Metric	Mean SCI-only	Mean treatment	Mean difference	t statistic	df	p.value	Cohens'd	Lower 95%CI	Upper 95%CI	Test	Normality Both	Variance p.value
Eg_Sparsity_0.38	0.688685	0.686399	0.00228606	2.507766191	6	0.046042	1.773258	5.55E-05	0.004517	Student's t-test (equal variances)	Yes	0.295433
Eg_Sparsity_0.40	0.699081	0.697159	0.00192143	2.46420205	6	0.04884	1.742454	1.35E-05	0.003829	Student's t-test (equal variances)	Yes	0.199435
Eg_Sparsity_0.41	0.704186	0.70276	0.001426	2.624700033	6	0.039338	1.855943	9.66E-05	0.002755	Student's t-test (equal variances)	Yes	0.482585
Eg_Sparsity_0.42	0.709299	0.708072	0.00122696	2.518477891	6	0.04538	1.780833	3.49E-05	0.002419	Student's t-test (equal variances)	Yes	0.45494
Eg_Sparsity_0.43	0.714459	0.713322	0.00113679	2.576415931	6	0.041971	1.821801	5.71E-05	0.002216	Student's t-test (equal variances)	Yes	0.199761
Eg_Sparsity_0.44	0.719547	0.718548	0.00099862	2.595123651	6	0.040929	1.83503	5.7E-05	0.00194	Student's t-test (equal variances)	Yes	0.160365
Eg_Sparsity_0.45	0.724613	0.723761	0.00085231	2.629129287	6	0.039105	1.859075	5.91E-05	0.001646	Student's t-test (equal variances)	Yes	0.170369
Eg_Sparsity_0.46	0.729716	0.729008	0.00070827	2.726454571	6	0.034347	1.927895	7.26E-05	0.001344	Student's t-test (equal variances)	Yes	0.153658
Eg_Sparsity_0.47	0.734813	0.734153	0.00066007	3.186941352	6	0.018909	2.253508	0.000153	0.001167	Student's t-test (equal variances)	Yes	0.071155
Eg_Sparsity_0.48	0.739874	0.73927	0.00060431	3.165889301	3.292531	0.044547	2.238622	2.63E-05	0.001182	Welch's t-test (unequal variances)	Yes	0.033668
Eg_Sparsity_0.49	0.744902	0.744365	0.00053777	3.11279573	3.219344	0.047993	2.201079	8.56E-06	0.001067	Welch's t-test (unequal variances)	Yes	0.022291

Metric	Mean SCI-only	Mean treatment	Mean difference	t statistic	df	p.value	Cohens'd	Lower 95%CI	Upper 95%CI	Test	Normality Both	Variance p.value
Eg_Sparsity_0.50	0.749935	0.74944	0.00049411	3.159304745	3.112086	0.048403	2.233966	6.37E-06	0.000982	Welch's t-test (unequal variances)	Yes	0.00839
Lp_Sparsity_0.37	1.463173	1.468367	-0.0051936	-2.488965	6	0.047228	-1.75996	-0.0103	-8.8E-05	Student's t-test (equal variances)	Yes	0.360244
Lp_Sparsity_0.38	1.452044	1.456885	-0.0048406	-2.50522451	6	0.046201	-1.77146	-0.00957	-0.00011	Student's t-test (equal variances)	Yes	0.290698
Lp_Sparsity_0.40	1.430451	1.434397	-0.0039462	-2.46125628	6	0.049036	-1.74037	-0.00787	-2.3E-05	Student's t-test (equal variances)	Yes	0.196538
Lp_Sparsity_0.41	1.420081	1.422963	-0.0028826	-2.6241573	6	0.039366	-1.85556	-0.00557	-0.00019	Student's t-test (equal variances)	Yes	0.479116
Lp_Sparsity_0.42	1.409843	1.412287	-0.0024439	-2.51785333	6	0.045419	-1.78039	-0.00482	-6.9E-05	Student's t-test (equal variances)	Yes	0.451996
Lp_Sparsity_0.43	1.399661	1.401893	-0.0022317	-2.57529734	6	0.042034	-1.82101	-0.00435	-0.00011	Student's t-test (equal variances)	Yes	0.198198
Lp_Sparsity_0.44	1.389764	1.391696	-0.0019323	-2.59402666	6	0.04099	-1.83425	-0.00376	-0.00011	Student's t-test (equal variances)	Yes	0.15923
Lp_Sparsity_0.45	1.380047	1.381673	-0.0016258	-2.62824127	6	0.039152	-1.85845	-0.00314	-0.00011	Student's t-test (equal variances)	Yes	0.169368
Lp_Sparsity_0.46	1.370396	1.371728	-0.0013318	-2.72545977	6	0.034393	-1.92719	-0.00253	-0.00014	Student's t-test (equal variances)	Yes	0.152876
Lp_Sparsity_0.47	1.36089	1.362114	-0.0012238	-3.18540377	6	0.018946	-2.25242	-0.00216	-0.00028	Student's t-test (equal variances)	Yes	0.070792

Metric	Mean SCI-only	Mean treatment	Mean difference	t statistic	df	p.value	Cohens'd	Lower 95%CI	Upper 95%CI	Test	Normality Both	Variance p.value
Lp_Sparsity_0.48	1.351581	1.352686	-0.0011051	-3.16437432	3.291558	0.044621	-2.23755	-0.00216	-4.7E-05	Welch's t-test (unequal variances)	Yes	0.033509
Lp_Sparsity_0.49	1.342458	1.343428	-0.0009701	-3.11148223	3.218704	0.048058	-2.20015	-0.00193	-1.5E-05	Welch's t-test (unequal variances)	Yes	0.022198
Lp_Sparsity_0.50	1.33345	1.334329	-0.0008793	-3.15808476	3.111796	0.048456	-2.2331	-0.00175	-1.1E-05	Welch's t-test (unequal variances)	Yes	0.008358

Table S4. Detailed statistical information on spinal cord DTI parameters with significant within-group differences.

FA, fractional anisotropy; RD, radial diffusivity; MD, mean diffusivity; AD, axial diffusivity.

Metric	Group	Time point (months)	Normality p.value	Test Type	t-statistic	p.value	Mean difference	SD difference	Lower 95%CI	Upper 95%CI	Cohen's d
FA	SCI-only	1	0.050105	Paired t-test	-3.82325	0.031504	-0.05691	0.029772	-0.10429	-0.00954	-1.91162
FA	SCI-only	3	0.060894	Paired t-test	-5.23032	0.013599	-0.11077	0.042356	-0.17817	-0.04337	-2.61516
FA	SCI-only	6	0.394046	Paired t-test	-7.59421	0.004738	-0.07009	0.018458	-0.09946	-0.04072	-3.79711
FA	SCI-only	12	0.252724	Paired t-test	-4.31223	0.022966	-0.09808	0.045489	-0.17046	-0.0257	-2.15611
FA	treatment	1	0.352392	Paired t-test	-6.53874	0.007271	-0.07232	0.02212	-0.10751	-0.03712	-3.26937

Table S5. Detailed statistical information on spinal cord DTI parameters with significant between-group differences.

FA, fractional anisotropy; RD, radial diffusivity; MD, mean diffusivity; AD, axial diffusivity.

Metric	Mean SCI-only	Mean treatment	Mean difference	t statistic	df	p.value	Cohens'd	Lower 95%CI	Upper 95%CI	Test	Normality Both	Variance p.value
FA	0.357122	0.46724063	-0.11012	-4.16402	6	0.005918	-2.9444	-0.17483	-0.04541	Student's t-test (equal variances)	Yes	0.898168
AD	1.86347275	1.15702887	0.706444	2.591088	6	0.041152	1.832176	0.039309	1.373579	Student's t-test (equal variances)	Yes	0.551842
MD	1.39484725	0.81656834	0.578279	2.724167	6	0.034452	1.926277	0.058855	1.097703	Student's t-test (equal variances)	Yes	0.360608
RD	1.1605345	0.64633807	0.514196	2.780464	6	0.031977	1.966085	0.061684	0.966708	Student's t-test (equal variances)	Yes	0.245584

Table S6. Detailed statistics from linear mixed-effects models testing the moderating role of regenerative therapy in the association between spinal cord DTI parameters and brain network metrics after SCI.

Eg, glocal efficiency; FA, fractional anisotropy; AUC, area under the curve; RSN, resting-state functional network.

Term	Global metric	Spinal cord DTI parameters	β	Std. Error	df	Pr(>|t|)
(Intercept)	RSN_Eg_Sparsity_0.1	FA	0.008381	0.090848	34.98654	0.927024
FA	RSN_Eg_Sparsity_0.1	FA	0.548831	0.236886	34.90553	0.026503
Group	RSN_Eg_Sparsity_0.1	FA	0.216361	0.115866	34.83794	0.070286
Time point	RSN_Eg_Sparsity_0.1	FA	0.002016	0.001759	29.77316	0.260782
FA:Group	RSN_Eg_Sparsity_0.1	FA	-0.56963	0.299494	34.61133	0.065525
(Intercept)	RSN_Eg_AUC	FA	-0.00111	0.045885	34.98987	0.980756
FA	RSN_Eg_AUC	FA	0.268561	0.119651	34.91424	0.031237
Group	RSN_Eg_AUC	FA	0.098258	0.058525	34.84451	0.102118
Time point	RSN_Eg_AUC	FA	0.001274	0.000889	29.65353	0.162322
FA:Group	RSN_Eg_AUC	FA	-0.26548	0.151287	34.6206	0.088144

Table S7. Detailed statistics from simple slopes analysis examining the association between spinal cord DTI parameters and brain network metrics after SCI, stratified by group.

Eg, glocal efficiency; FA, fractional anisotropy; AUC, area under the curve; RSN, resting-state functional network.

Global metric	Spinal cord DTI parameters	Group	β	SE	df	Lower 95%CI	Upper 95%CI	p.value
RSN_Eg_Sparsity_0.1	FA	SCI-only	0.54883091	0.24932999	34.90644703	0.042615623	1.055046198	0.034424921
RSN_Eg_Sparsity_0.1	FA	treatment	-0.02079454	0.174683117	32.91217719	-0.376226044	0.334636963	0.905966122
RSN_Eg_AUC	FA	SCI-only	0.268561	0.125977	34.91742	0.012791	0.52433	0.040135
RSN_Eg_AUC	FA	treatment	0.003085	0.08828	32.93879	-0.17653	0.182705	0.972331

Table S8. Detailed statistics from paired comparison of slopes on spinal cord DTI parameters-brain network metrics associations between the SCI-only and treatment groups.

Eg, glocal efficiency; FA, fractional anisotropy; AUC, area under the curve; RSN, resting-state functional network.

Global metric	Spinal cord DTI parameters	Contrast	β	SE	df	Lower 95%CI	Upper 95%CI	p.value
RSN_Eg_Sparsity_0.1	FA	SCI-only - treatment	0.569625	0.313347	34.61507	-0.06676	1.206006	0.077747
RSN_Eg_AUC	FA	SCI-only - treatment	0.265475	0.158332	34.63454	-0.05608	0.587027	0.102605

Table S9. Variance explained and effect sizes from linear mixed effects models of spinal cord DTI parameters × group interactions on brain network metrics. Eg, glocal efficiency; FA, fractional anisotropy; AUC, area under the curve; RSN, resting-state functional network.

Global metric	Spinal cord DTI parameters	Marginal R^2	Conditional R^2	Cohen's f^2 (interaction)
RSN_Eg_Sparsity_0.1	FA	0.132201116	0.207101342	0.189120462
RSN_Eg_AUC	FA	0.139640127	0.212048124	0.17772005

Table S10. Detailed statistics from linear mixed-effects models testing the moderating role of regenerative therapy in the association between brain network metrics and locomotor parameters after SCI.

Eg, glocal efficiency; λ, normalized characteristic path length; Eloc, local efficiency; Cp, clustering coefficient.; AUC, area under the curve; RSN, resting-state functional network; SCN, structural covariance network.

Term	Locomotor parameters	Global metric	β	Std. Error	df	Pr(>|t|)
(Intercept)	Path length	SCN_Eloc_Sparsity_0.10	-7.31065	2.96837	10.63884	0.032192
SCN_Eloc_Sparsity_0.10	Path length	SCN_Eloc_Sparsity_0.10	9.765872	3.840715	10.63818	0.027982
Group	Path length	SCN_Eloc_Sparsity_0.10	8.857132	4.167417	22.35636	0.044842
Time point	Path length	SCN_Eloc_Sparsity_0.10	0.017133	0.006816	20.51405	0.020392
SCN_Eloc_Sparsity_0.10:Group	Path length	SCN_Eloc_Sparsity_0.10	-11.3691	5.410599	22.43572	0.047063
(Intercept)	Path length	SCN_Eg_Sparsity_0.10	3.179543	0.90953	26.78722	0.001664
SCN_Eg_Sparsity_0.10	Path length	SCN_Eg_Sparsity_0.10	-6.13714	1.888811	26.80485	0.003111
Group	Path length	SCN_Eg_Sparsity_0.10	-4.29128	1.995402	26.96663	0.040633
Time point	Path length	SCN_Eg_Sparsity_0.10	0.01812	0.005775	21.37392	0.0049
SCN_Eg_Sparsity_0.10:Group	Path length	SCN_Eg_Sparsity_0.10	9.087161	4.14069	26.97021	0.036984
(Intercept)	Path length	SCN_Eg_AUC	10.64539	3.276049	26.981	0.003092
SCN_Eg_AUC	Path length	SCN_Eg_AUC	-37.689	11.8495	26.98145	0.003675
Group	Path length	SCN_Eg_AUC	-15.4624	7.059951	26.65017	0.037448
Time point	Path length	SCN_Eg_AUC	0.018381	0.005848	21.56265	0.004806
SCN_Eg_AUC:Group	Path length	SCN_Eg_AUC	56.24339	25.55318	26.65087	0.036584
(Intercept)	Path length	SCN_Cp_Sparsity_0.10	-3.28231	1.163217	10.14491	0.017869
SCN_Cp_Sparsity_0.10	Path length	SCN_Cp_Sparsity_0.10	6.287683	2.072843	10.13443	0.012423
Group	Path length	SCN_Cp_Sparsity_0.10	4.719447	1.767887	25.23598	0.013094
Time point	Path length	SCN_Cp_Sparsity_0.10	0.016057	0.006705	21.58622	0.025765
SCN_Cp_Sparsity_0.10:Group	Path length	SCN_Cp_Sparsity_0.10	-8.30283	3.1765	25.37472	0.014849

Term	Locomotor parameters	Global metric	β	Std. Error	df	Pr(>|t|)
(Intercept)	Path length	SCN_Cp_AUC	-3.57091	1.880137	13.10427	0.079762
SCN_Cp_AUC	Path length	SCN_Cp_AUC	13.74678	6.773095	13.10496	0.063209
Group	Path length	SCN_Cp_AUC	6.96325	2.856039	24.10866	0.022506
Time point	Path length	SCN_Cp_AUC	0.014065	0.006384	21.98737	0.03837
SCN_Cp_AUC:Group	Path length	SCN_Cp_AUC	-24.7977	10.29608	24.15663	0.024004
(Intercept)	Path length	SCN_λ_Sparsity_0.10	-2.71777	0.854735	26.69147	0.003714
SCN_λ_Sparsity_0.10	Path length	SCN_λ_Sparsity_0.10	2.645289	0.764303	26.71157	0.001824
Group	Path length	SCN_λ_Sparsity_0.10	7.49949	2.883068	26.87927	0.014919
Time point	Path length	SCN_λ_Sparsity_0.10	0.016509	0.005587	21.45242	0.007449
SCN_λ_Sparsity_0.10:Group	Path length	SCN_λ_Sparsity_0.10	-6.66681	2.596011	26.87554	0.016106
(Intercept)	Path length	SCN_λ_AUC	-8.16426	2.316923	26.38196	0.001573
SCN_λ_AUC	Path length	SCN_λ_AUC	17.97397	4.960952	26.38536	0.001219
Group	Path length	SCN_λ_AUC	19.84487	7.836037	26.69796	0.017522
Time point	Path length	SCN_λ_AUC	0.017377	0.005596	21.45265	0.005265
SCN_λ_AUC:Group	Path length	SCN_λ_AUC	-42.34	16.79525	26.69857	0.017991
(Intercept)	Step height	RSN_Eg_AUC	0.009785	0.013165	26.83712	0.463785
RSN_Eg_AUC	Step height	RSN_Eg_AUC	0.023491	0.122482	25.02742	0.849455
Group	Step height	RSN_Eg_AUC	-0.08359	0.041544	15.90231	0.061479
Time point	Step height	RSN_Eg_AUC	0.001041	0.000713	22.22736	0.158693
RSN_Eg_AUC:Group	Step height	RSN_Eg_AUC	0.845605	0.393373	16.35037	0.046879
(Intercept)	Stride length	SCN_Cp_Sparsity_0.10	-1.26459	0.794413	12.66638	0.136054
SCN_Cp_Sparsity_0.10	Stride length	SCN_Cp_Sparsity_0.10	2.669101	1.415693	12.66522	0.082526
Group	Stride length	SCN_Cp_Sparsity_0.10	2.626787	1.132619	25.40031	0.028705
Time point	Stride length	SCN_Cp_Sparsity_0.10	0.003266	0.004073	21.50132	0.431332

Term	Locomotor parameters	Global metric	β	Std. Error	df	Pr(>|t|)
SCN_Cp_Sparsity_0.10:Group	Stride length	SCN_Cp_Sparsity_0.10	-4.5528	2.033488	25.52857	0.034106

Table S11. Detailed statistics from simple slopes analysis examining the association between brain network metrics and locomotor parameters after SCI, stratified by group.

Eg, glocal efficiency; λ, normalized characteristic path length; Eloc, local efficiency; Cp, clustering coefficient.; AUC, area under the curve; RSN, resting-state functional network; SCN, structural covariance network.

Locomotor parameters	Global metric	Group	β	SE	df	Lower 95%CI	Upper 95%CI	p.value
Path length	SCN_Eloc_Sparsity_0.10	SCI-only	9.765872	4.279547	12.37305	0.472625	19.05912	0.040917
Path length	SCN_Eloc_Sparsity_0.10	treatment	-1.60326	4.018062	25.17332	-9.87573	6.669204	0.693248
Path length	SCN_Eg_Sparsity_0.10	SCI-only	-6.13714	2.051374	26.79336	-10.3477	-1.92655	0.005892
Path length	SCN_Eg_Sparsity_0.10	treatment	2.950022	3.974068	26.99019	-5.20423	11.10427	0.464306
Path length	SCN_Eg_AUC	SCI-only	-37.689	12.80276	26.9805	-63.9589	-11.419	0.006594
Path length	SCN_Eg_AUC	treatment	18.55443	24.64991	26.48158	-32.0694	69.17824	0.458259
Path length	SCN_Cp_Sparsity_0.10	SCI-only	6.287683	2.280622	10.13208	1.215105	11.36026	0.020005
Path length	SCN_Cp_Sparsity_0.10	treatment	-2.01515	2.449582	22.03746	-7.09477	3.064472	0.41952
Path length	SCN_Cp_AUC	SCI-only	13.74678	7.557781	12.7794	-2.60951	30.10307	0.092429
Path length	SCN_Cp_AUC	treatment	-11.0509	7.987835	25.5747	-27.4835	5.381584	0.178478
Path length	SCN_λ_Sparsity_0.10	SCI-only	2.645289	0.831097	26.68929	0.93909	4.351488	0.003685
Path length	SCN_λ_Sparsity_0.10	treatment	-4.02152	2.64811	26.79416	-9.45695	1.413905	0.140566
Path length	SCN_λ_AUC	SCI-only	17.97397	5.422174	26.34271	6.835586	29.11236	0.002675
Path length	SCN_λ_AUC	treatment	-24.366	17.47035	26.7011	-60.231	11.49893	0.1746
Step height	RSN_Eg_AUC	SCI-only	0.023491	0.127786	25.24744	-0.23956	0.286541	0.855617
Step height	RSN_Eg_AUC	treatment	0.869096	0.424395	15.29772	-0.03395	1.772141	0.058145
Stride length	SCN_Cp_Sparsity_0.10	SCI-only	2.669101	1.572352	12.60718	-0.73855	6.07675	0.114127
Stride length	SCN_Cp_Sparsity_0.10	treatment	-1.8837	1.487451	21.76331	-4.97043	1.203034	0.21876

Table S12. Detailed statistics from paired comparison of slopes on brain network metrics-locomotor parameters associations between the SCI-only and treatment groups.

Eg, glocal efficiency; λ, normalized characteristic path length; Eloc, local efficiency; Cp, clustering coefficient.; AUC, area under the curve; RSN, resting-state functional network; SCN, structural covariance network.

Locomotor parameters	Global metric	Contrast	β	SE	df	Lower 95%CI	Upper 95%CI	p.value
Path length	SCN_Eloc_Sparsity_0.10	SCI-only - treatment	11.36913	5.832935	23.34898	-0.68724	23.42551	0.063393
Path length	SCN_Eg_Sparsity_0.10	SCI-only - treatment	-9.08716	4.475625	26.96845	-18.2709	0.096566	0.052288
Path length	SCN_Eg_AUC	SCI-only - treatment	-56.2434	27.82373	26.63335	-113.37	0.882988	0.053392
Path length	SCN_Cp_Sparsity_0.10	SCI-only - treatment	8.302833	3.322529	25.37415	1.465069	15.1406	0.019279
Path length	SCN_Cp_AUC	SCI-only - treatment	24.79773	11.1094	24.03135	1.870628	47.72482	0.035187
Path length	SCN_λ_Sparsity_0.10	SCI-only - treatment	6.66681	2.775557	26.86586	0.970506	12.36311	0.023493
Path length	SCN_λ_AUC	SCI-only - treatment	42.34001	18.28675	26.67738	4.79745	79.88257	0.028532
Step height	RSN_Eg_AUC	SCI-only - treatment	-0.8456	0.437025	17.15762	-1.767	0.075791	0.069662
Stride length	SCN_Cp_Sparsity_0.10	SCI-only - treatment	4.552798	2.146462	25.51651	0.13661	8.968985	0.0438

Table S13. Variance explained and effect sizes from linear mixed effects models of brain network metric × group interactions on locomotor parameters. Cohen's f^2 was calculated to quantify the effect size of the interaction terms. Cohen's f^2 is calculated by comparing the marginal R^2 of the full model, which includes all variables, with that of a reduced model from which the target variable has been excluded. Negative values arise when the full model accounts for less variance than the reduced model [1,2].

Locomotor parameters	Global metric	Marginal R^2	Conditional R^2	Cohen's f^2 (interaction)
Path length	SCN_Eloc_Sparsity_0.10	0.343338	0.391729	0.028487
Path length	SCN_Eg_Sparsity_0.10	0.40423	0.52126	0.006616
Path length	SCN_Eg_AUC	0.395846	0.517842	-0.01518
Path length	SCN_Cp_Sparsity_0.10	0.399371	0.436099	0.121425
Path length	SCN_Cp_AUC	0.335424	0.467297	0.061116
Path length	SCN_λ_Sparsity_0.10	0.449905	0.547465	0.117885
Path length	SCN_λ_AUC	0.46307	0.544489	0.061554
Step height	RSN_Eg_AUC	0.25411	0.276757	-0.07123
Stride length	SCN_Cp_Sparsity_0.10	0.319871	0.433879	0.249079

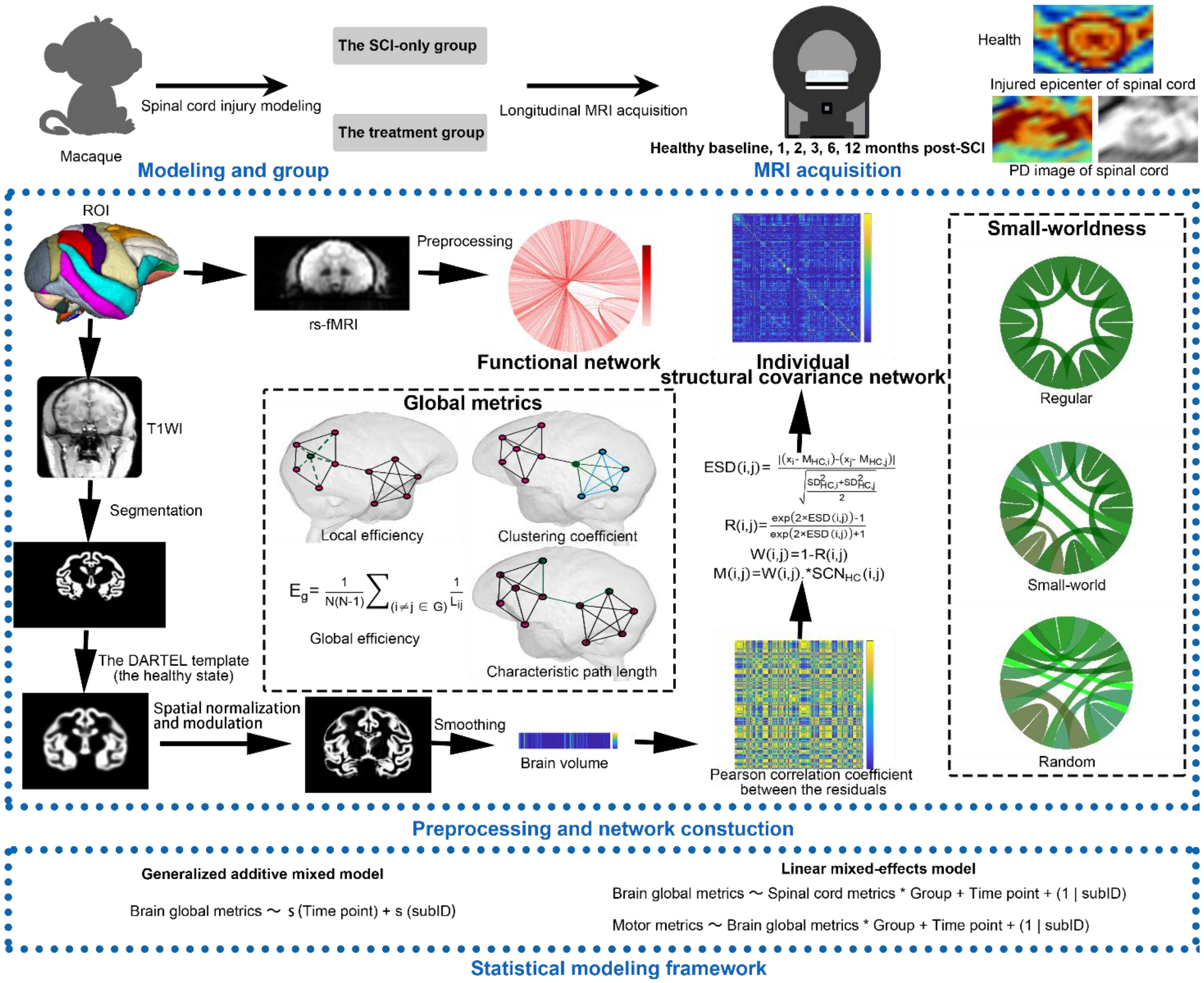

Macaque
Spinal cord injury modeling
The SCI-only group
The treatment group
Longitudinal MRI acquisition
Healthy baseline, 1, 2, 3, 6, 12 months post-SCI
Health
Injured epicenter of spinal cord
PD image of spinal cord
Modeling and group
MRI acquisition
ROI
rs-fMRI
Preprocessing
Functional network
Individual structural covariance network
Small-worldness
Regular
Small-world
Random
T1WI
Segmentation
The DARTEL template (the healthy state)
Spatial normalization and modulation
Smoothing
Brain volume
Pearson correlation coefficient between the residuals
Global metrics
Local efficiency
Clustering coefficient
Global efficiency
Characteristic path length
$E_g = \frac{1}{N(N-1)} \sum_{(i \neq j \in G)} \frac{1}{L_{ij}}$
$ESD(i,j) = \frac{|(x_i - M_{HC,i}) - (x_j - M_{HC,j})|}{\sqrt{\frac{SD^2_{HC,i} + SD^2_{HC,j}}{2}}}$
$R(i,j) = \frac{\exp(2 \times ESD(i,j)) - 1}{\exp(2 \times ESD(i,j)) + 1}$
$W(i,j) = 1 - R(i,j)$
$M(i,j) = W(i,j) .* SCN_{HC}(i,j)$
Preprocessing and network constuction
Generalized additive mixed model
Brain global metrics ~ s (Time point) + s (subID)
Linear mixed-effects model
Brain global metrics ~ Spinal cord metrics * Group + Time point + (1 | subID)
Motor metrics ~ Brain global metrics * Group + Time point + (1 | subID)
Statistical modeling framework

Figure S1. Experimental design, data processing, and statistical analysis pipeline.

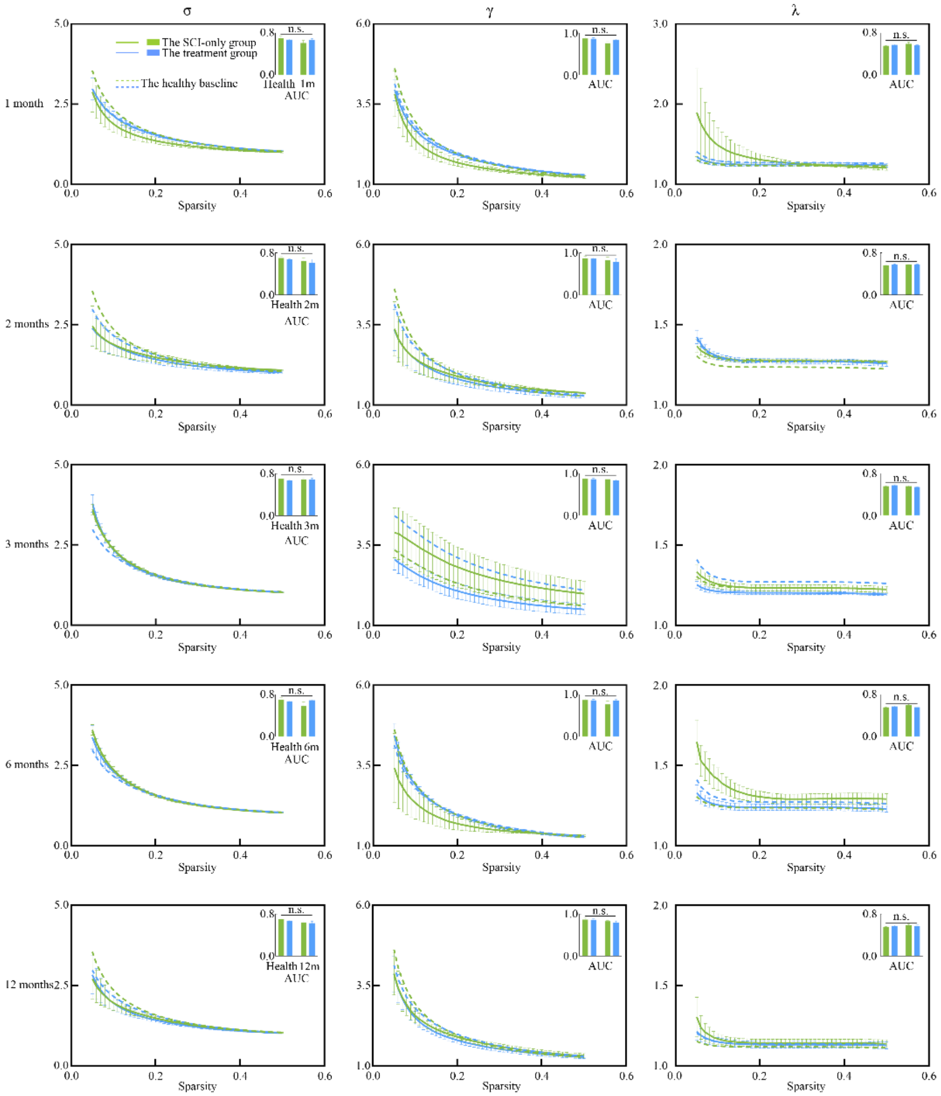
σ
γ
λ
The SCI-only group
The treatment group
The healthy baseline
1 month
2 months
3 months
6 months
12 months
Sparsity
AUC
n.s.
Health 1m
Health 2m
Health 3m
Health 6m
Health 12m

Figure S2. Between-group and within-group differences in small-world characteristics within functional network, including σ, γ and λ.

Solid squares represent significant differences between the SCI-only group and its healthy baseline. Open squares represent significant differences between the treatment group and its healthy baseline. Solid circles represent significant differences between the SCI-only group and the treatment group. A p-value less than 0.05 is considered to indicate significant between-group differences. n.s., none significance. γ , normalized clustering coefficient; λ, normalized characteristic path length; σ, small-worldness.

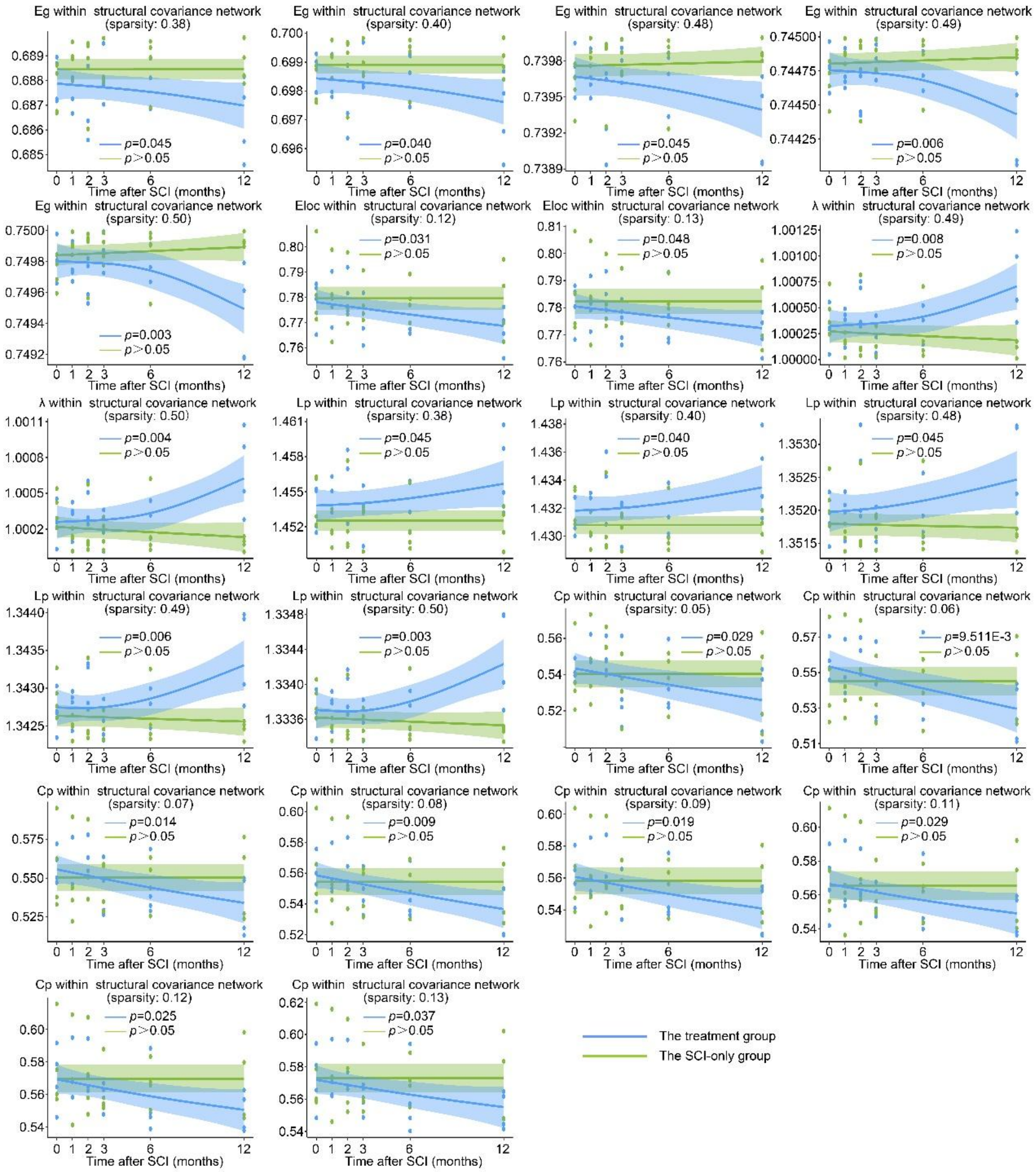
Eg within structural covariance network (sparsity: 0.38)
p=0.045
p>0.05
Eg within structural covariance network (sparsity: 0.40)
p=0.040
p>0.05
Eg within structural covariance network (sparsity: 0.48)
p=0.045
p>0.05
Eg within structural covariance network (sparsity: 0.49)
p=0.006
p>0.05
Eg within structural covariance network (sparsity: 0.50)
p=0.003
p>0.05
Eloc within structural covariance network (sparsity: 0.12)
p=0.031
p>0.05
Eloc within structural covariance network (sparsity: 0.13)
p=0.048
p>0.05
λ within structural covariance network (sparsity: 0.49)
p=0.008
p>0.05
λ within structural covariance network (sparsity: 0.50)
p=0.004
p>0.05
Lp within structural covariance network (sparsity: 0.38)
p=0.045
p>0.05
Lp within structural covariance network (sparsity: 0.40)
p=0.040
p>0.05
Lp within structural covariance network (sparsity: 0.48)
p=0.045
p>0.05
Lp within structural covariance network (sparsity: 0.49)
p=0.006
p>0.05
Lp within structural covariance network (sparsity: 0.50)
p=0.003
p>0.05
Cp within structural covariance network (sparsity: 0.05)
p=0.029
p>0.05
Cp within structural covariance network (sparsity: 0.06)
p=9.511E-3
p>0.05
Cp within structural covariance network (sparsity: 0.07)
p=0.014
p>0.05
Cp within structural covariance network (sparsity: 0.08)
p=0.009
p>0.05
Cp within structural covariance network (sparsity: 0.09)
p=0.019
p>0.05
Cp within structural covariance network (sparsity: 0.11)
p=0.029
p>0.05
Cp within structural covariance network (sparsity: 0.12)
p=0.025
p>0.05
Cp within structural covariance network (sparsity: 0.13)
p=0.037
p>0.05
Time after SCI (months)
The treatment group
The SCI-only group

Figure S3. The longitudinal trajectories of global metrics with significant changes with the duration of injury at sparsity range of 0.05-0.09 and 0.11-0.50. λ, normalized characteristic path length; Eg, global efficiency; Lp, characteristic path length; Eloc, local efficiency; Cp, clustering coefficient.

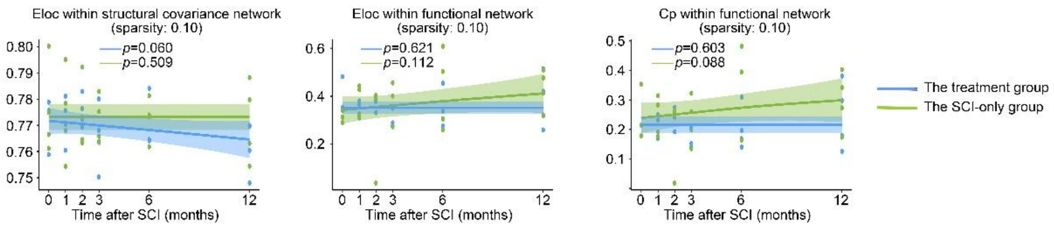

Figure S4. The longitudinal trajectories of global metrics that exhibit a downward or upward trend with the duration of injury at network sparsity thresholds of 0.10.

Eloc, local efficiency; Cp, clustering coefficient.

References:

1. LaHuis, D.M.; Hartman, M.J.; Hakoyama, S.; Clark, P.C. Explained Variance Measures for Multilevel Models. *Organizational Research Methods* **2014**, *17*, 433-451, doi:10.1177/1094428114541701.
2. Snijders, T.; Bosker, R. Multilevel Analysis: An Introduction to Basic and Advanced Multilevel Modeling. *http://lst-iiep.iiep-unesco.org/cgi-bin/wwwi32.exe/[in=epidoc1.in]/?t2000=013777/(100)* **1999**.
